# Pragmatic Language Disorder in Parkinson’s Disease and the Potential Effect of Cognitive Reserve

**DOI:** 10.3389/fpsyg.2019.01220

**Published:** 2019-06-19

**Authors:** Sonia Montemurro, Sara Mondini, Matteo Signorini, Anna Marchetto, Valentina Bambini, Giorgio Arcara

**Affiliations:** ^1^Department of General Psychology, University of Padua, Padua, Italy; ^2^Human Inspired Technology Research Centre, University of Padua, Padua, Italy; ^3^Gruppo Veneto Diagnostica e Riabilitazione, Padua, Italy; ^4^Center for Neurocognition, Epistemology and Theoretical Syntax, University School of Advanced Studies IUSS, Pavia, Italy; ^5^IRCCS San Camillo Hospital, Venice, Italy

**Keywords:** Parkinson’s disease, pragmatic abilities, Cognitive Reserve, communication, discourse, figurative language

## Abstract

It is known that patients with Parkinson’s Disease (PD) may show deficits in several areas of cognition, including speech and language abilities. One domain of particular interest is pragmatics, which refers to the capacity of using language in context for a successful communication. Several studies showed that some specific aspects of pragmatics – both in production and in comprehension – might be impaired in patients with PD. However, a clear picture of pragmatic abilities in PD is still missing, as most of the existing studies focused on specific aspects of the pragmatic competence rather than on sketching a complete pragmatic profile. Moreover, little is known on the potential role of protective factors in compensating the decline of communicative skills as the disease progresses. The present study has two aims: (1) to provide a complete picture of pragmatic abilities in patients with PD, by using a comprehensive battery (Assessment of Pragmatic Abilities and Cognitive Substrates, APACS) and by investigating the relationship with other aspects of cognitive functioning (e.g., working memory and Theory of Mind) and (2) to investigate whether Cognitive Reserve, i.e., the resilience to cognitive impairment provided by life experiences and activities, may compensate for the progressive pragmatic deficits in PD. We found that patients with PD, compared to healthy matched controls, had worse performance in discourse production and in the description of scenes, and that these impairments were tightly correlated with the severity of motor impairment, suggesting reduced intentionality of engaging in a communicative exchange. Patients with PD showed also an impairment in comprehending texts and humor, suggesting a problem in inferring from stories, which was related to general cognitive impairment. Notably, we did not find any significant difference between patients and controls in figurative language comprehension, a domain that is commonly impaired in other neurodegenerative diseases. This might be indicative of a specific profile of pragmatic impairment in patients with PD, worth of further investigation. Finally, Cognitive Reserve measures showed a high degree of association with pragmatic comprehension abilities, suggesting that the modification of life-styles could be a good candidate for compensating the possible problems in understanding the pragmatic aspects of language experienced by patients with PD.

## Introduction

Parkinson’s Disease (PD) is a progressive neurodegenerative disorder that affects the dopaminergic neurons of the substantia nigra, in the ventral midbrain. Specifically, the degeneration of nigral neurons reduces the dopamine availability for neurotransmission in the corpus striatum and such biochemical imbalance results in the typical motor symptoms of patients with PD (e.g., bradykinesia, muscular rigidity, resting tremor, and postural instability; Litvan et al., 2003; Barone et al., 2009). Patients with PD commonly show also non-motor symptoms, such as sleep disorders, mood disturbances, and, importantly, cognitive dysfunctions. Interestingly, some of these non-motor symptoms can even precede the manifestation of the motor ones (Chaudhuri et al., 2006).

With regard to cognitive symptoms, patients with PD show several deficits as the disease progresses, mostly presenting executive dysfunctions, visuospatial difficulties and memory difficulties, sometimes generating the symptomatic patterns of dementia (Kehagia et al., 2010; Biundo et al., 2014). PD cognitive impairment can arise independently from motor symptoms and has gained increasing importance during the last decade (Barone et al., 2009; Kehagia et al., 2010). The efficiency of executive functions, in particular, can be strongly affected by the striatal brain damage, which causes a disruption to the frontal-striatal circuitry. Executive deficits of PD have been extensively investigated in the literature (Morris et al., 1988; Owen et al., 1992; Owen, 1997), showing that patients may have specific difficulties in cognitive planning, as well as in phonemic and in semantic fluency tests, two tasks associated with executive abilities. Interestingly, it has been argued that the executive impairment in PD may underlie, or may be strongly associated with a series of difficulties in pragmatic abilities (Holtgraves and McNamara, 2010).

Pragmatic abilities refer to the human capability to communicate in different contexts, with different interlocutors, recognizing and expressing communicative intentions (Grice, 1975; Bara, 2010). The crucial role of pragmatic abilities is especially evident whenever there is a major discrepancy between the more basic (literal) meaning and the intended meaning. This is the case of expressions such as metaphors, idioms, irony, or proverbs. For example, in the phrase: “At the end, John decided to bite the bullet,” the intended meaning cannot be inferred from the literal meaning of the words forming the phrase. To understand the communicative intention, the phrase must be processed at a higher cognitive level, allowing to grasp the sense of the utterance. In other words, pragmatic abilities rely on a high-order interplay of cognitive functions supporting context-dependent language processing (Martin and McDonald, 2003).

Neurological diseases may impair pragmatic abilities, generating communication disorders in daily social interaction. Importantly, different neurological diseases may lead to different pragmatic profiles, suggesting that different diseases can affect the “pragmatic system” at different levels (Martin and McDonald, 2003). Despite reported evidence that also patients with PD may show a pragmatic language disorder, a clear neuropsychological picture of the pragmatic profile of PD is still missing.

### Pragmatic Abilities in Neurological Disorders and Parkinson’s Disease

Traditionally, disorders in communication and pragmatics have been associated with damages in the right-hemisphere or with specific pathological conditions, such as traumatic brain injury (TBI) and schizophrenia.

Right hemisphere damage (RHD) was one of the first clinical conditions associated with pragmatic impairment. Patients with RHD can show several pragmatic difficulties (Joanette et al., 1990; McDonald, 2000) in both comprehension and production, including problems in understanding metaphors (Winner and Gardner, 1977) and communicative intentions in deceit and irony tasks (Parola et al., 2016). Also patients with TBI often show pragmatic deficits, which have been largely documented especially with respect to discourse production. For example, through analysis of micro- and macro-linguistic aspects of discourse, TBI patients were shown to fail at the level of discourse informativeness, and this impairment was associated with executive dysfunction and with problems in Theory of Mind (Marini et al., 2014; Bosco et al., 2017). In the case of schizophrenia, a long tradition of studies has evidenced the presence a pragmatic impairment (for recent developments, see Bambini et al., 2016a; Parola et al., 2018), with major difficulties in several aspects of communication (DeLisi, 2001; Bosco and Parola, 2017), as well as in understanding figurative expressions and humor (Brüne and Bodenstein, 2005; Kiang et al., 2007) Also for schizophrenia, pragmatic impairment has been described as tied to deficits in cognition and social-cognition (Bambini et al., 2016a).

Nowadays it is acknowledged that a pragmatic impairment can be found in a large number of neurological and even neurodevelopmental disorders (see Cummings, 2014, 2017; Cappelli et al., 2018). Focusing on neurodegenerative diseases, pragmatic deficits seem widespread. For instance, Amanzio et al. (2008) measured the ability to understand figurative language of Alzheimer (AD) patients by using a metaphor comprehension task. Patients with AD had poor understanding of non-conventional (novel) metaphors, and this impairment was associated with executive deficits. Recently, a broader pragmatic impairment has been described in the case of patients with amyotrophic lateral sclerosis (ALS), who can show pragmatic difficulties both in the production and in the comprehension modalities, with main failures in maintaining the discourse topic, over- or under-informativeness, and problems in solving non literal meanings (Bambini et al., 2016b). Problems have been reported also in Multiple Sclerosis (MS; Carotenuto et al., 2018a) with quite strong associations with Theory of Mind abilities.

Importantly for the aims of this study, several studies highlighted a pragmatic impairment also in patients with PD (McNamara and Durso, 2003; Holtgraves and Giordano, 2017). Disruption of pragmatic abilities in PD has been for long investigated in relation with attentional, short-term memory, and executive deficits, and often traced back to these more basic impairments (e.g., Grossman et al., 1992; McKinlay et al., 2009). Beside more general cognitive functions, PD is often associated with other relevant deficits in language as verbal fluency, voice articulation (Ho et al., 1999), and verb inflection, which could be also at the core of a pragmatic impairment. Additionally, patients with PD may show difficulties in other paralinguistic abilities crucial for understanding communicative intentions, such as understanding emotions from faces and recognizing facial expressions. Beside these impairments, some studies have reported more specific pragmatic difficulties in patients with PD, in terms of reduced spontaneous speech production (Illes et al., 1988), poor conversational appropriateness, prosody impairment or slowness in processing speed. However, the literature still lacks a comprehensive description of the pragmatic profile characteristic of PD. Moreover, some crucial aspects that could be associated with pragmatic abilities in PD have been neglected, in particular Theory of Mind (Martin and McDonald, 2003), which was linked to pragmatic disruption in other neurodegenerative diseases (e.g., Carotenuto et al., 2018a). Finally, and crucially from a clinical perspective, little is known about those factors that might help to maintain pragmatic abilities in patients with PD as the disease progresses. In this context, one of the best candidates is Cognitive Reserve, which has been already proved to modulate the progression of other degenerative disorders like Alzheimer’s Disease (Roe et al., 2007), MS (Sumowski et al., 2009), and Huntington’s disease (Bonner-Jackson et al., 2013).

### The Potential Effect of Cognitive Reserve on Pragmatic Abilities in Parkinson’s Disease

Cognitive Reserve is a concept developed to describe the evidence that higher amount of lifetime exposures to educational, occupational attainment, and leisure activities allows to better tolerate worsening of cognitive functioning caused by neurodegeneration. In particular, individuals with greater reserve are more able to cope with the neuronal degeneration, and would manifest the symptom of the disease later, compared with individuals who have lower reserve (Roe et al., 2007). The interest about the preventive role of Cognitive Reserve is increasingly growing, providing evidence about the necessity of maintaining an active and socially rich life-style, and of keeping cognitive functioning efficient over time (see Barulli and Stern, 2013 and Cabeza et al., 2018 for further explanations).

With regard to language, Cognitive Reserve has been shown to predict verbal comprehension skills and vocabulary size enhancement in healthy older persons (Schaie, 1989; Christensen et al., 1997; Arbuckle et al., 1998) but little is still known about the role of Cognitive Reserve in the higher-level domain of pragmatic communication. There are several reasons why focusing on Cognitive Reserve is of great relevance. First, this would help us to understand how pragmatic abilities are supported and interlaced with life styles. A second reason is related to the clinical consequences: if Cognitive Reserve is strictly associated with pragmatic performance, then, whenever pragmatic abilities are clinically assessed in PD, the Cognitive Reserve should be taken into account too. Finally, being Cognitive Reserve responsible for different rates of behavioral changes along disease progression, if a strong relationship between Cognitive Reserve and pragmatic abilities is present, this suggests that pragmatic skills could be preserved by promoting behaviors that enrich the reserve itself.

In the specific case of patients with PD, Cognitive Reserve has been shown to have beneficial effects on their global cognitive performance, and especially in executive function tasks. However, this is not always the case, as some studies failed in showing beneficial impact of Cognitive Reserve on cognitive functioning in PD. It is not known whether Cognitive Reserve can predict pragmatic abilities in patients with PD and whether possible beneficial effects of Cognitive Reserve are homogenous or not across distinct aspects of pragmatics. Indeed, the beneficial effect of having high Cognitive Reserve is not a clear-cut phenomenon, and it varies depending on the nature of the function involved in a task. For example, math abilities in aging seem to be related only to some specific aspects of Cognitive Reserve, i.e., education but scarcely related to Cognitive Reserve, as in the case of proper name retrieval (Arcara et al., 2017; Montemurro et al., 2018).

### Aims of the Study

The present study has two main goals.

The first is to sketch the profile of pragmatic performance in patients with PD by means of the APACS test (Assessment of Pragmatic Abilities and Cognitive Substrates). APACS is a standardized test that assesses discourse production and non-literal meaning comprehension by means of six tasks, allowing for a comprehensive evaluation of participants’ pragmatic skills. This would fill the gap about what type of pragmatic profile can be expected in the case of patients with PD, furthermore allowing to compare the pragmatic profile of PD with the profile of other neurological populations assessed with the same test. The pragmatic assessment was complemented by a series of tests associated with cognitive variables relevant for pragmatics (i.e., the general cognitive profile, Theory of Mind, verbal comprehension, and working memory). Based on the literature, we hypothesized a generalized impairment in pragmatic abilities. In particular, in line with previous works with APACS and neurodegenerative diseases (Bambini et al., 2016b; Carotenuto et al., 2018a), we expected a worse performance in patients with PD as compared to healthy controls, especially in tasks on figurative language.

The second goal of this study is to understand to what extent the decline of pragmatic abilities in PD could be prevented by high Cognitive Reserve. In our study, Cognitive Reserve was measured by means of a semi-structured interview (Cognitive Reserve Index questionnaire, CRIq), which takes into account not only the level of education, but also the frequency and the cognitive load required in working activities^1^ and the frequency of leisure time activities, in patients and healthy controls. To the best of our knowledge, this is the first study investigating the possible effect of Cognitive Reserve on the ability to perform pragmatic tasks in patients with PD. Assuming that both pragmatic abilities and Cognitive Reserve are fundamentally developed and enhanced through social life experiences, and that higher Cognitive Reserve is typically associated with better cognitive performance, the hypothesis was that higher Cognitive Reserve would be associated with better pragmatic abilities. Moreover, we expected the relationship with Cognitive Reserve to be stronger for pragmatic aspects such as figurative language understanding, compared with discourse production, given the reported effects of education on non-literal speech comprehension (Champagne-Lavau et al., 2012).

## Methods

### Participants

A series of 47 consecutive patients with PD coming for neurological rehabilitation after initial diagnosis were recruited at the *Gruppo Veneto Diagnostica e Riabilitazione* (Padua, Italy). We excluded patients who had clinically severe cardiovascular, metabolic, and psychiatric diseases or neurosurgical procedures (including deep brain stimulation). We also enrolled a sample of 45 healthy controls matched, as much as possible, with the patient group. All participants were monolingual native speakers of Italian.

Patients with PD had a mean Age of 72.00 years (SD = 7.36) and a mean Education of 10.36 years (SD = 4.82), 12 were females and 35 males. The healthy controls had a mean Age of 70.47 (SD = 7.50) and a mean Education of 10.42 (SD = 4.65), 13 were females and 32 males. Between-group differences on these variables were not significant at *t*-tests [age: *t*(90) = -0.98, *p* = 0.32; education: *t*(90) = 0.06, *p* = 0.95]. As a more robust test for matching of demographic variables we also employed the Test of Equivalence, which assesses if two groups can be considered significantly equivalent. Specifically, in a Test of Equivalence the null hypothesis is the presence of a difference of at least a specified amount (with a threshold set *a priori*). We found that patients with PD and healthy controls were significantly equivalent for Age when the threshold was 5 years (tost *p* = 0.014), and significantly equivalent for Education with a threshold of 2 years (tost *p* = 0.03). Finally, the two groups were not different in terms of proportion of female/male participants [χ^2^ (1) = 0.01, *p* = 0.89, with Yates correction]. Concerning clinical variables, patients had a mean Unified Parkinson Disease Rating Scale (UPDRS-III) score of 34.52 (SD = 13.30), a mean Hoehn and Yahr (H&Y) score of 2.15 (SD = 0.94), and a mean number of Years from Onset of the disease of 7.5 (SD = 3.86). The patients with PD showed different degrees of cognitive impairment: using a stratification based on MoCA scores (Biundo et al., 2014), our sample was composed of 31 patients with PD without cognitive disorders (PD-CNT, MoCA > 25), 10 patients with PD with Mild Cognitive Impairment (PD-MCI, MoCA > 20 and ≤25), and 6 patients with PD Dementia (PDD, MoCA < 20). A table including the details of each patient is reported in the Supplementary Table S1.

The study was approved by the ethics committee of the University of Padua (Italy). All participants signed an informed consent explaining the nature of the study. The research was completed in accordance with the Declaration of Helsinki.

### Assessment

Patients were assessed for clinical characteristics of the diseases, pragmatic abilities, general cognitive abilities, and Cognitive Reserve.

#### Demographic and Clinical Assessment

Two main demographic variables were considered in the analysis: Age, as number of years, and Education, as number of successfully completed years of school and university/ college courses.

For the patients with PD, three additional clinical variables were included.

Two variables measured the severity of extrapyramidal symptoms, which was evaluated with the H&Y (Hoehn and Yahr, 1967) and with the motor UPDRS-III (Fahn et al., 1987). The H&Y system is based on a scale from 1 to 5, with higher scores indicating a more severe impairment, which can be associated with a variety of neurocognitive issues such as depression or dementia, and poorer quality of life. The motor UPDRS-III ranges from 0 to 108 and classifies the severity of the disease based on tremor, slowness (bradykinesia), stiffness (rigidity) and balance, with higher scores suggesting a more severe impairment. Finally, duration of illness was considered and quantified as number of Years from Onset (after first diagnosis).

#### Pragmatic Assessment

**FIGURE 1:**
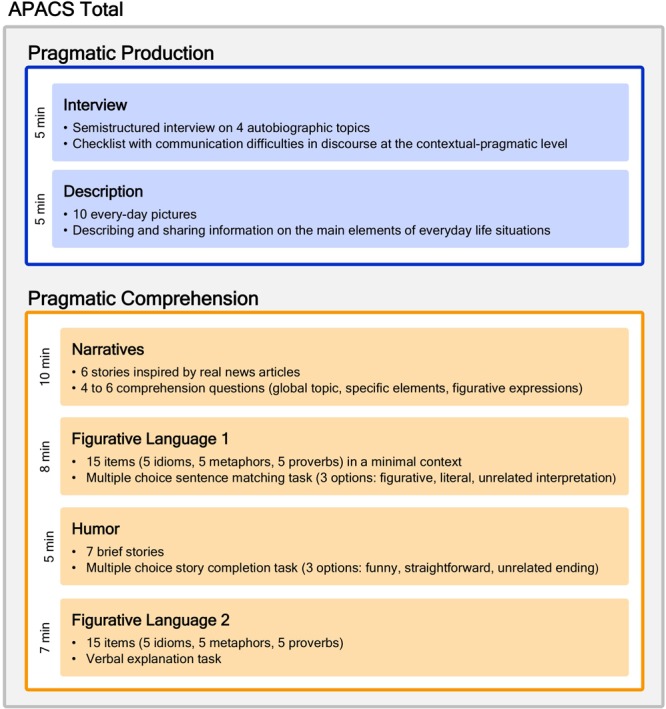
The APACS test. The figure shows the structure of the APACS test. It consists of two sections: Production (in blue), which encompasses two tasks, and Comprehension (in orange), which encompasses four tasks. Adapted from Arcara and Bambini (2016).

All participants were administered the APACS (Arcara and Bambini, 2016). APACS is a test that investigates pragmatic skills in both expressive and receptive modalities through six tasks (see Figure 1), two dedicated to production (Interview and Description) and four dedicated to comprehension (Narratives, Figurative Language 1, Humor, Figurative Language 2).

From the theoretical point of view, the APACS test is grounded in Neo-Gricean pragmatics, assuming that communication involves not only coding and decoding, but rather it is a rational, cooperative activity (Grice, 1975), involving also inferential processes to derive the speaker’s meaning and to link different information in the text, as well as the ability to meet the interlocutor’s needs (Sperber and Wilson, 2005). More specifically, inferential abilities are especially evident when there is a gap between the literal and the intended meaning, as in figurative language, and in the textual dimension, when inferences are needed to bridge different aspects of a text. Conversely, the ability to meet the interlocutor’s need is especially evident in conversation, as reflected in skills such as providing the appropriate amount of information, taking verbal initiative when appropriate, etc. The tasks included in APACS are intended to evaluate these different aspects of the pragmatic competence. In details, the Interview and the Description tasks measure the ability to meet the interlocutor’s needs in conversation, both in a discourse on autobiographical topics and in everyday contexts. The Narrative task measures comprehension and inferential abilities at the discourse level, while Figurative Language (1 and 2) and Humor task focus on the inferential skills to understand non-literal meanings.

APACS shows satisfactory psychometric properties and is provided with normative data for the Italian population. Concerning the performance of the general population, in all comprehension tasks of APACS there is a negative effect of age (the higher the age, the lower the performance) and a positive effect of education (the higher the education, the higher the performance). A negative effect of age is found also for the Interview task of Production. Even if all APACS tasks can be considered easy to perform, they show a certain degree of variability also in healthy participants, and have no marked ceiling effect (see Arcara and Bambini, 2016).

##### Task 1: Interview (production section)

This task measures discourse organization and engagement in conversation. Participant undergoes a semi-structured interview based on autobiographical topics, in which communication difficulties are measured. The frequency of each type of communication difficulty is reported (i.e., always/sometimes/never) and then converted into scores (0/1/2). Various dimensions of discourse are rated, mostly at the pragmatic level: speech (e.g., repetition, incomplete utterances, echolalia), informativeness (over- or under-informativeness, loss of verbal initiative), information flow (e.g., missing referents, wrong order of the discourse elements, abrupt topic shift), paralinguistic dimensions (e.g., altered intonation, loss of eye-contact, fixed facial expression, abuse of gesture). Errors in grammar and vocabulary are also annotated, as they affect the communicative effectiveness of the discourse. Maximal score: 44.

##### Task 2: Description (production section)

This task measures the ability of producing and sharing information of everyday life situations, based on the description of photographs that represent scenes of daily life (e.g., children playing in a park). The examiner assigns a score for each salient element in the picture by differentiating missed identification, partially correct identification, correct identification (0/1/2). Maximal score: 48.

##### Task 3: Narratives (comprehension section)

This task measures the ability to understand the main aspects of a narrative text. A series of stories inspired by real newspaper, radio, and TV news are read to the participants, and followed by comprehension questions on explicit and implicit contents, with the latter based on inferential processes. Each question is scored for accuracy (either 0/1 or 0/1/2). Maximal score: 56.

##### Task 4: Figurative Language 1 (comprehension section)

This task measures the ability to infer non-literal meanings through multiple-choice questions following the presentation of idioms, novel metaphors, and proverbs. Each item is scored either 1 or 0 according to the accuracy. Maximal score: 15.

##### Task 5: Humor (comprehension section)

This task measures the ability to comprehend verbal humor through multiple-choice questions. The participant is asked to select the best punch line of a story. Each item is scored either 1 or 0 according to the accuracy. Maximal score: 7.

##### Task 6: Figurative Language 2 (comprehension section)

This task measures the ability to infer non-literal meanings through verbal explanation of idioms, novel metaphors, and proverbs. The maximal score of 2 is given to an item when the subject provides a good description of the meaning of the figurative expression; a score of 1 is given when the participant provides an incomplete explanation, such as concrete examples, but fails in providing an abstract meaning; a score of 0 is given when the participant provides a literal explanation, paraphrases the figurative expression or does not know it. Maximal score: 30.

##### Composite scores

Three composite pragmatic scores are derived from the six APACS single task scores. The Pragmatic Production score is calculated from Interview and Description, whereas the Pragmatic Comprehension score is calculated from Narratives, Figurative Language 1, Figurative Language 2, and Humor. Each composite score is obtained by transforming the original task scores in proportion and averaging these proportions. Thus, each task equally contributes to the final composite score. Moreover, the APACS Total score is calculated by averaging Pragmatic Production and Pragmatic Comprehension scores.

#### General Cognitive Assessment

All participants were administered a series of tasks both on global functioning and on specific cognitive functions. Given the limited available time to test each participant, assessment was restricted only to some relevant tests.

##### MoCA

The MoCA test (Nasreddine et al., 2005; Conti et al., 2015) is a brief neuropsychological screening consisting of eight sub-tests assessing different cognitive domains (i.e., memory, language, visuospatial skills, executive functions, and orientation in time and space). The MoCA is widely used in the clinical practice and is very sensitive to mild cognitive impairment in aging, especially in neurodegenerative diseases. Its administration lasts about 10 min. Maximal score: 30.

##### Token Test

To examine language comprehension, the 36-item-version of the Token Test was administered (De Renzi and Vignolo, 1962). The participant is required to perform some actions in response to simple verbal commands. This test detects receptive language disorders. All commands consist of not redundant words referring to different tokens, which are circles and rectangles in different colors and sizes. Maximal score: 36.

##### Digit Span Backward

To examine working memory ability, participants were administered the Digit Span Backward task. In this task each item consists of a list of digits. Participants are required to immediately repeat the list of digits in reverse order (Monaco et al., 2013). After each list, if the subject has succeeded in repeating it, another list one digit longer is presented. If the subject has failed, a second list of the same length is presented. If the participant is successful on the second item list, a list one digit longer is given, as before. However, if the participant fails on the second list too, the test is ended. The length of the digit sequences gradually increases starting with a sequence of three numbers to a sequence of maximum eight items. The span is established as the length of the longest list recalled correctly. Maximal score: 8.

##### Theory of Mind

To evaluate Theory of Mind, we used the Story-Based Empathy Task (SET; Dodich et al., 2015), which is a non-verbal test developed for the assessment of intention and emotion attribution in neurodegenerative diseases. The total SET score is the result of performance in 18 stimuli, grouped into three conditions that assess the ability to infer others’ intentions (Story-Based Empathy Task: Intention Attribution, SET-IA) and emotions (Story-Based Empathy Task: Emotion Attribution, SET-EA), compared to a control condition testing causal inference (Story-Based Empathy Task: Causal Inference, SET-CI). Each condition is made of six trials in which the participant is asked to select the correct ending of a sequence of pictures. A score of 1 is assigned only for the selection of the correct ending, and the global score is computed based on the total of correct answers. Maximal score of each subtask: 6, maximum total score: 18.

#### Cognitive Reserve Assessment

Cognitive Reserve was measured with the CRIq (Nucci et al., 2012), consisting in a semi-structured interview. CRIq requires about 10 min to be completed and includes twenty questions grouped into three sections: Education (CRI-Education), Working activities (CRI-WorkingActivity), and Leisure time activities (CRI-LeisureTime). CRI-Education is made up of years of formal education and any additional training courses lasted at least 6 months. CRI-WorkingActivity refers to the cognitive load and personal responsibility of an occupation, combined with the number of years for which the occupation has been carried out for a minimum of 5 years. Finally, CRI-LeisureTime measures the frequency and the amount of intellectual, social, and physical activities (e.g., reading newspapers or books, playing music, participation in charitable activities, traveling, doing sports, etc.) carried out for a minimum of 5 years. Additionally, the questionnaire includes items about life-long experiences that require a certain cognitive load (e.g., years of bank account management). The CRI Total score is an estimation of Cognitive Reserve. It is the average of the three subscores standardized and transposed to a scale with a mean of 100 and a standard deviation of 15 (see Nucci et al., 2012 for details). The CRI Total score can be classified into five ordered levels: Low (less than 70), Medium–low (70–84), Medium (85–114), Medium–high (115–130) and High (more than 130).

### Data Analysis

First, we compared the performance of patients with PD and healthy controls in the APACS test and in the other neuropsychological tests (MoCa, Token, Digit Span Backward, SET) by means of separate independents *t*-tests, with Cohen’s *d* as measure of effect size, and *p*-values adjusted with false discovery rate (FDR) correction (Benjamini and Hochberg, 1995).

To analyze individual performance, APACS scores of each patient were compared to cut-off values calculated as 5th percentile of the healthy control matched sample (for an analogous approach, see Bambini et al., 2016b). Additional analyses restricted to PD-CNT patients (i.e., patients without cognitive impairment as measured by MoCA, Biundo et al., 2014) are reported in the Supplementary Material.

Furthermore, we performed two analyses to evaluate which scores, among Demographic and Clinical variables, General Cognitive variables, and Cognitive Reserve, were mostly associated with the performance in APACS: a correlation analysis and a Random Forest analysis. In the Correlation analysis we used Pearson’s correlations corrected with FDR. We also compared the correlation coefficients between PD patients and healthy controls with the test for difference of independent correlations as implemented in the *r.pair* function of the *psych* R package (Revelle, 2017).

Random Forests is a method from machine learning, which allows to predict values of a variable from a large set of other variables. We fitted two Random Forests, one with the Pragmatic Production score as dependent variable and one with the Pragmatic Comprehension score as dependent variable. In both Random Forests the predictors were: Demographic and Clinical variables (i.e., Age, Education, UPDRS-III, H&Y, Years from Onset), General Cognitive variables (i.e., MoCA, Token Test, Digit Span Backward, SET), and Cognitive Reserve. Random Forests were calculated using unbiased partitioning as implemented in the *cforest* function included in the *party* package (Strobl et al., 2009a,b). Each forest consisted of 500 trees. All statistical analyses were performed with R, release 3.3.2 (R Core Team, 2016). Details on the Random Forest analysis are reported in the Supplementary Material.

## Results

Descriptive statistics and *t*-tests comparing patients with PD and healthy controls in all administered tests are reported in Tables 1, 2.

**Table 1:** Descriptive statistics and results for *t*-tests comparing patients with PD and healthy controls in the pragmatic assessment.

APACS task or composite score	Mean Parkinson (SD)	Mean Controls (SD)	df	*t*	Cohen’s *d*	*p*-Value
Interview	35.09 (6.15)	38.82 (2.45)	90	–3.8	–0.79	<0.001
Description	37.26 (5.85)	46.4 (1.99)	90	–9.9	–2.1	<0.001
Narratives	42.87 (8.25)	49.6 (4.33)	90	–4.9	–1	<0.001
Figurative Language 1	12.91 (1.87)	13.24 (2.09)	90	–0.8	–0.17	0.48
Humor	5.21 (1.94)	6.09 (1.24)	90	–2.6	–0.53	0.015
Figurative Language 2	20.38 (5.66)	21.09 (4.04)	90	–0.69	–0.14	0.49
Pragmatic Production	0.78 (0.1)	0.92 (0.03)	90	–9	–1.9	<0.001
Pragmatic Comprehension	0.77 (0.15)	0.83 (0.1)	90	–2.6	–0.54	0.015
APACS Total	0.77 (0.11)	0.88 (0.06)	90	–5.9	–1.2	<0.001

*The table shows the results of *t*-tests comparing patients with PD and controls on the APACS tasks, on the APACS composite scores, and the APACS Total. The first column reports the name of the APACS task, the composite scores and the APACS Total. The second and the third columns report the mean (and SD) of patients with PD and healthy controls. The fourth column reports the degrees of freedom associated with the *t*-test; the fifth column reports the *t*-values; the sixth column reports the Cohen’s *d*. The seventh column reports the *p*-values (corrected with FDR method).*

**Table 2:** Descriptive statistics and results for *t*-test comparing patients with PD and healthy controls in all neuropsychological tests.

Test	Mean Parkinson (SD)	Mean Controls (SD)	df	*t*	Cohen’s *d*	*p*-value
MoCA	25.4 (4.55)	25.18 (2.56)	90	0.29	0.061	0.77
Token	33.11 (2.97)	33.92 (1.69)	89	–1.6	–0.33	0.11
Digit SPAN BW	3.65 (1.4)	4.47 (0.94)	89	–3.2	–0.68	0.0017
SET TOT	13.71 (2.99)	16.02 (1.5)	88	–4.6	–0.97	<0.001
SET IA	4.62 (1.25)	5.44 (0.72)	88	–3.8	–0.8	<0.001
SET EA	4.62 (1.28)	5.31 (0.82)	88	–3	–0.63	0.003
SET CI	4.44 (1.37)	5.16 (0.98)	88	–2.8	–0.59	0.006
CRI-Education	102.6 (17.14)	104.36 (16.1)	88	–0.5	–0.1	0.62
CRI-WorkingAct	106.91 (23.32)	104.91 (16.8)	88	0.47	0.097	0.64
CRI-LeisureTime	104.67 (24.22)	124.91 (17.07)	88	–4.6	–0.96	<0.001
CRI-Tot	107.62 (21.38)	115.13 (19.21)	88	–1.8	–0.37	0.083

*The table shows the results of *t*-tests comparing patients with PD and healthy controls on the neuropsychological tests taken into account in our study. The first column reports the name of the test, tasks, or scale. The second and third columns report the mean (and SD) of patients with PD and healthy controls. The fourth column reports the degrees of freedom associated with the *t*-test; the fifth column reports the *t*-values; the sixth column reports the Cohen’s *d*. The seventh column reports the *p*-values (corrected with FDR method). Test Legend: SET-Tot, Story Empathy Test-Total; SET-IA, Story Empathy Test-Intention Attribution; SET-EA Story Empathy Test-Emotion Attribution; SET-CI, Story Empathy Test-Causal Inference; CRI-Educaton, Cognitive Reserve Index-Education section; CRI-WorkingAct, Cognitive Reserve Index-Working Activities; CRI-LeisureTime, Cognitive Reserve Index-Leisure Time; CRI-Tot, Cognitive Reserve Index-Total.*

**FIGURE 2:**
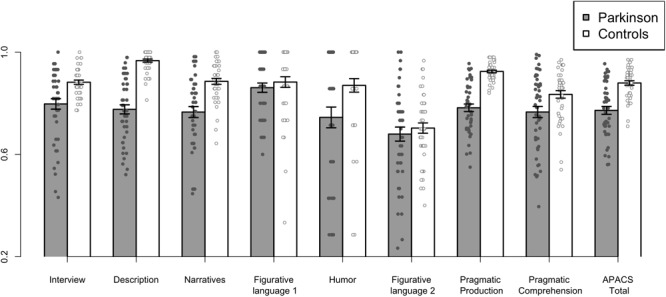
Performance of patients with PD and Controls in Pragmatic tasks and composite scores. The figure shows the performance of patients with PD and healthy controls in the APACS tasks and in the composite scores, i.e., Pragmatic Production, Pragmatic Comprehension, and APACS Total. All raw scores were transformed in proportion (relative to the maximum obtainable score) before plotting. Gray bars indicate the mean performance of patients with PD, whereas white bars indicate the mean performance of healthy controls. The small circles denote the scores for each participant (a small jitter was added in the x-axis to improve visibility of individual scores).

The group analysis comparing the patients with PD and healthy controls in the APACS scores showed significant differences in the Interview, Description, Narratives, Humor, Pragmatic Production, Pragmatic Comprehension, and APACS Total composite scores (FDR correction; *p*s < 0.05). The highest effect size was found for Description (Cohen’s *d* = 2.1). Detailed results can be found in Table 1. No significant differences between patients with PD and controls were observed in the two tasks assessing figurative language comprehension (i.e., Figurative Language 1 and Figurative Language 2). A barplot showing the results of patients with PD and controls in APACS scores is reported in Figure 2.

The results of the comparison between patients with PD and healthy controls in neuropsychological tests are reported in Table 2. Patients with PD had a significantly lower performance than healthy controls in the Digit Span Backward, SET-Tot, SET-IA, SET-EA, SET-CI, and CRI-LeisureTime.

We also performed an additional comparison on APACS performance, restricted to the 31 patients with PD without clear signs of cognitive impairment (PD-CNT). This analysis yielded a similar profile, showing a significant lower performance than controls in Interview, Description, Narratives, and Pragmatic Production. Differing from the complete sample, here no difference in performance compared to healthy controls was observed in Humor, Pragmatic Comprehension, and APACS Total (for details, see Supplementary Material).

### Individual Performance in APACS Tasks in Patients With PD

**FIGURE 3:**
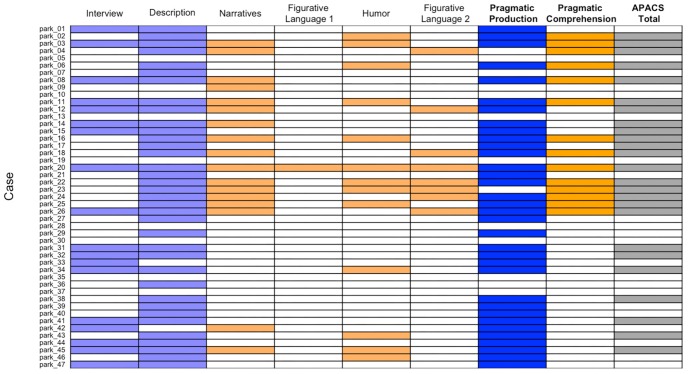
Performance below cut-off of Parkinson patients in pragmatic tasks and composite scores. The figure shows patients with PD who scored below cut-off (i.e., below 5th percentile of healthy control data) in the APACS tasks and in the three composite scores. Each row denotes a patient, whose case number is reported in the left part of the figure, consistently with Supplementary Table S1. Each column denotes a task or composite score. White cells indicate a performance equal to or above cut-off, whereas colored cells indicate a performance below cut-off. Light blue cells are used in the columns with the pragmatic tasks included in the Pragmatic Production score and dark blue cells are used for the Pragmatic Production score. Light orange cells are used in the columns of the pragmatic tasks included in the Pragmatic Comprehension score, and dark orange cells are used for the Pragmatic Comprehension score. Dark gray is used for APACS Total.

To better assess individual performance, separately for each patient, we took into account whether the score in each APACS task and composite score was below normative cut-off. Several patients showed a performance below cut-off, especially in production tasks (i.e., Interview and Description) and in the Pragmatic Production composite score, but performance below cut-off was widespread (Number of Patients below cut-off/Total number of patients: Interview: 18/47; Description: 36/47; Narratives: 17/47 Figurative Language 1: 1/47; Humor: 13/47, Figurative Language 2: 8/47; Pragmatic Production: 34/47, Pragmatic Comprehension: 14/47; APACS Total: 25/47) (see Figure 3).

### Pairwise Correlation Analysis Between Pragmatic and Neuropsychological Tests in Patients With PD and Controls

Pairwise correlations between APACS scores and neuropsychological variables were performed separately for patients with PD and healthy controls. Detailed results on the correlations are reported in Table 3 (for patients with PD and healthy controls). Scatterplots based on all the pairwise relationships are reported in the Supplementary Material.

**Table 3:** Correlations in patients and healthy controls, between APACS tasks and variables from Clinical Assessment.

(A) Patients with Parkinson’s Disease																	
	Age	Edu	UPDRS-III	H&Y	Years FromOnset	MoCA	Token Test	Digit Span BW	SET-Tot	SET-IA	SET-EA	SET-CI	CRI-Education	CRI-Working Act	CRI-Leisure Time	CRI-Tot
Interview	0	0.22	–0.49 ^∗^	–0.46^∗^	0	0.26	0.26	0.18	0.07	–0.03	0.04	0.15	0.26	0.3	0.16	0.29
Description	–0.23	0.15	–0.29	–0.2	–0.14	0.29	0.38^∗^	0.17	0.41^∗^	0.23	0.28	0.46^∗^	0.14	0.16	0.13	0.18
Narratives	–0.09	0.51^∗^	0.08	–0.16	–0.28	0.49^∗^	0.48^∗^	0.24	0.47^∗^	0.29	0.42^∗^	0.41^∗^	0.51^∗^	0.55^∗^	0.36^∗^	0.56^∗^
Figurative Language 1	–0.14	0.66^∗^	0	0.01	–0.15	0.41^∗^	0.49^∗^	0.11	0.51^∗^	0.24	0.45^∗^	0.5^∗^	0.59^∗^	0.6^∗^	0.3	0.55^∗^
Humor	–0.12	0.43^∗^	–0.06	–0.06	–0.02	0.34^∗^	0.52^∗^	0.31	0.41^∗^	0.17	0.48^∗^	0.31	0.35^∗^	0.35^∗^	0.37^∗^	0.47^∗^
Figurative Language 2	–0.11	0.62^∗^	0.1	–0.02	–0.06	0.55^∗^	0.56^∗^	0.14	0.48^∗^	0.21	0.54^∗^	0.39^∗^	0.55^∗^	0.49^∗^	0.37^∗^	0.55^∗^
Pragmatic Production	–0.27	0.17	–0.57^∗^	–0.49^∗^	–0.03	0.33^∗^	0.36^∗^	0.17	0.26	0.07	0.15	0.38^∗^	0.17	0.25	0.09	0.21
Pragmatic Comprehension	–0.09	0.68^∗^	0.05	–0.08	–0.17	0.55^∗^	0.67^∗^	0.27	0.59^∗^	0.3	0.57^∗^	0.5^∗^	0.63^∗^	0.55^∗^	0.49^∗^	0.67^∗^
APACS Total	–0.21	0.56^∗^	–0.24	–0.29	–0.15	0.55^∗^	0.63^∗^	0.29	0.52^∗^	0.22	0.47^∗^	0.52^∗^	0.51^∗^	0.51^∗^	0.39^∗^	0.57^∗^
**(B) Healthy controls**																
	**Age**	**Edu**	**MoCA**	**Token Test**	**Digit Span BW**	**SET-Tot**	**SET-IA**	**SET-EA**	**SET-CI**	**CRI-Education**	**CRI-WorkingAct**	**CRI-LeisureTime**	**CRI-Tot**

Interview	–0.3	0.37^∗^	0.48^∗^	0.38^∗^	0.29	0.18	0.13	–0.01	0.19	0.29	0.35^∗^	0.48^∗^	0.43^∗^
Description	–0.3	–0.11	0.16	0.07	–0.09	0.17	0.3	0.08	–0.1	–0.17	–0.02	–0.02	–0.08
Narratives	–0.43^∗^	0.29	0.72^∗^	0.47^∗^	0.32	0.47^∗^	0.43^∗^	0.13	0.28	0.18	0.3	0.29	0.3
Figurative Language 1	–0.26	0.57^∗^	0.54^∗^	0.41^∗^	0.51^∗^	0.35^∗^	0.21	0.11	0.26	0.5^∗^	0.41^∗^	0.46^∗^	0.53^∗^
Humor	–0.29	0.03	0.22	0.29	0.31	0.22	0.49^∗^	–0.12	–0.01	–0.02	0	0.08	0.03
Figurative Language 2	–0.25	0.46^∗^	0.64^∗^	0.41^∗^	0.42^∗^	0.54^∗^	0.2	0.41^∗^	0.33	0.43^∗^	0.54^∗^	0.52^∗^	0.57^∗^
Pragmatic Production	–0.41^∗^	0.25	0.47^∗^	0.39^∗^	0.19	0.24	0.26	0.05	0.11	0.15	0.29	0.39^∗^	0.32
Pragmatic Comprehension	–0.4^∗^	0.43^∗^	0.65^∗^	0.51^∗^	0.53^∗^	0.49^∗^	0.44^∗^	0.15	0.25	0.35^∗^	0.38^∗^	0.44^∗^	0.45^∗^
APACS Total	–0.44^∗^	0.41^∗^	0.68^∗^	0.53^∗^	0.48^∗^	0.49^∗^	0.44^∗^	0.15	0.25	0.32	0.39^∗^	0.47^∗^	0.45^∗^

*General Cognitive Assessment and Cognitive Reserve. The table reports the Pearson’s *r*-values for the correlation among APACS tasks and scores and all the other variables included in the study. Asterisks (^∗^) denotes correlation with *p* < 0.05 after FDR multiple comparison correction. Test Legend: SET-Tot, Story Empathy Test-Total; SET-IA, Story Empathy Test-Intention Attribution; SET-EA Story Empathy Test-Emotion Attribution; SET-CI, Story Empathy Test-Causal Inference; CRI-Education, Cognitive Reserve Index-Education section; CRI-WorkingAct, Cognitive Reserve Index-Working Activities; CRI-LeisureTime, Cognitive Reserve Index-Leisure Time; CRI-Tot, Cognitive Reserve Index-Total.*

Both patients with PD and healthy controls showed several significant correlations.

Concerning the Demographic variables, Education was positively associated with several APACS scores both in patients with PD and controls, while Age showed only a few significant negative correlations. With regard to the Clinical variables (available only for patients with PD), both UPDRS-III and H&Y were negatively correlated with Interview scores and APACS Production scores. Years from Onset did not show any significant correlation.

Overall, variables belonging to General Cognitive Assessment showed several significant correlations both in patients with PD and in healthy controls, with a qualitatively similar pattern. These correlations involved especially the comprehension tasks of APACS (i.e., Narratives, Figurative Language 1, Humor, Figurative Language 2, and consequently Pragmatic Comprehension) and were rarely significantly associated with production scores (i.e., Interview, Description, and Pragmatic Production).

Finally, all Cognitive Reserve scores showed several significant correlations, both in patients with PD and in healthy controls, again involving especially the comprehension tasks of APACS.

The analysis that compared the difference in correlations between patients with PD and healthy controls did not show any significant difference, confirming a similarity in the pattern of the two groups. Detailed results of these contrasts are reported (both with corrected and with uncorrected *p*-values) in Supplementary Material.

Given the high number of significant correlations, to better understand these results, we run two exploratory Principal Component Analyses (PCA), not planned from the beginning of the study, one for patients with PD and one for healthy controls. These PCA included only a subset of the relevant scores: MoCA, Token Test, SET-Total, CRI Total, and Pragmatic Comprehension score.^2^ The PCA on patients with PD showed that a single component was able to explain 62% of the variability in all scores, while the PCA on controls showed that a single component was able to explain 52%. Both these results suggest a common underlying association across all the variables. Details on the PCA are reported in the Supplementary Material.

### Prediction of Pragmatic Performance in Patients With PD With Random Forest Analysis

The results of Random Forests are interpreted by inspecting the Importance associated with each predictor in the model. Importance indicates how much each model worsens (i.e., lowers the ability to predict data) if a given variable is taken out from the model. It is crucial to remember that Importance in Random Forests cannot be considered as the relevance of a variable alone, but it should rather be considered as the relevance of that variable in (potentially very complex) interactions with the other variables.

**FIGURE 4:**
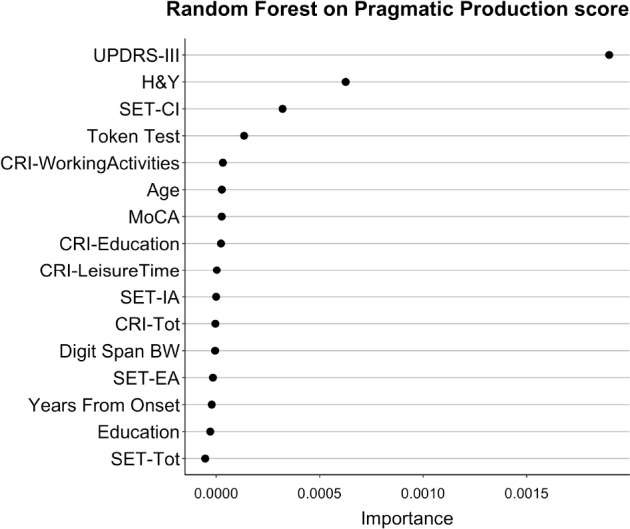
Importance of all variables in Random Forests with Pragmatic Production as dependent variable. The figure shows the Importance associated with each variable in the Random Forests with Pragmatic Production score from APACS as dependent variable. Variables are sorted from top to bottom according to Importance, so that the variables on top are the ones with the highest Importance.

In the Random Forest with Pragmatic Production as dependent variable, the most important predictors were: UPDRS-III, H&Y, SET-CI, and Token Test. The Importance of all predictors for this model is shown in Figure 4 (Random Forest *R*^2^ = 0.33).

**FIGURE 5:**
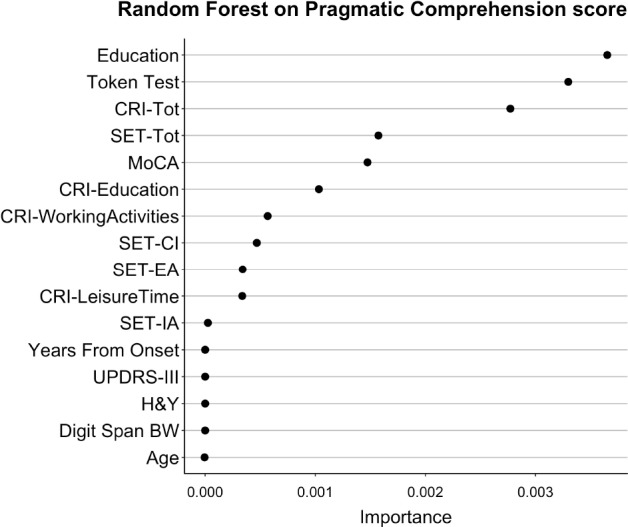
Importance of all variables in Random Forests with Pragmatic Comprehension as dependent variable. The figure shows the Importance associated with each variable in the Random Forests with Pragmatic Comprehension score from APACS as dependent variable. Variables are sorted from top to bottom according to Importance, so that the variables on top are the ones with the highest Importance.

In the Random Forest with Pragmatic Comprehension as dependent variable, the most important predictors were: Education, Token Test, CRI-Total, and SET-Tot. The Importance of all predictors for this model is shown in Figure 5 (Random Forest *R*^2^ = 0.61).

## Discussion

In the present study we investigated the pragmatic profile of patients with PD using a standardized assessment tool (APACS; Arcara and Bambini, 2016) that evaluates pragmatic skills in both the production and the comprehension modalities. In our analyses we took into account also other relevant variables, such as the severity of motor symptoms, the global cognitive functioning, working memory, ToM abilities and verbal comprehension skills. Moreover, we investigated the potential influence of Cognitive Reserve as a protective factor from decline of pragmatic abilities in patients with PD.

At the group level, patients with PD showed a worse performance than matched healthy controls in several pragmatic tasks of APACS: Interview, Description, Narratives, and Humor. These differences in performance were also reflected in the three APACS composite scores: Pragmatic Comprehension, Pragmatic Production, and APACS Total. Interestingly, patients with PD had a performance that was not significantly different from controls in Figurative Language 1 and Figurative Language 2, two tasks that assess (via forced choices and via open responses, respectively) the ability to understand figurative language expressions. We also evaluated the difference at group level in other clinical, neuropsychological, and Cognitive Reserve tests. Our analysis revealed significant differences in Digit Span, some subscales of SET (Story-based Empathy Test), and in CRI-LeisureTime.

The pattern of results on pragmatic abilities was not only investigated at group level but also at individual level, inspecting the number of patients who scored below cut-off, defined as 5th percentile of the control sample. Results showed that 82% of patients with PD fell below cut-off in at least one pragmatic task, with 53% of patients presenting an overall pragmatic language disorder, which we defined as APACS Total score below cut-off. Importantly, these results indicate that not only patients with PD have a group performance different from the healthy controls, but that many of them are likely to show an impaired performance, when assessed in clinical contexts.

Concerning the performance on APACS Production tests, a qualitative inspection of the results showed that in Interview, a semi-structured clinical interview with a checklist of specific communicative behaviors, patients’ performance was mainly impaired in discourse organization, with a lack of important details in the produced speech; similar problems were found in the Description task, where subjects are required to provide information about photographs showing everyday life scenes. In this latter task, patients seemed to be able to describe the picture, but often failed to spontaneously convey salient elements needed for a complete explanation of the depicted scene (i.e., the main elements, such as the location or the agent). In general, these problems in production are in line with previous studies, in which patients with PD showed major deficits in discourse organization, in terms of under-informativeness, turn-taking disruption, inappropriate levels of politeness, and paralinguistic deficits (Illes, 1989; McNamara and Durso, 2003; Saldert et al., 2014; Holtgraves and Giordano, 2017). This qualitative inspection was confirmed by a *post hoc* exploratory analysis that was performed by comparing patients with PD and controls, separately on each item of the Interview task of APACS. This analysis showed that patients with PD often did not report the main referents of the discourse, lacked verbal initiative, showed impairment in paralinguistic aspects (e.g., fixed facial expression), and often made grammatical mistakes (see Supplementary Table S6).

Furthermore, both correlations and Random Forests analyses pointed to a very clear-cut relationship of the production abilities of patients with PD with their motor symptoms, as measured by the UPDRS-III and H&Y scales. This is in line with previous findings where patients with PD showed increasing impaired speech as the disease progressed (Ho et al., 1999; Hall et al., 2011), and also suggests that motor symptoms are related to discourse aspects beyond mere articulation. It is important to stress that this relationship does not imply that worse scores on pragmatic production are due to articulation problems: indeed, the discourse production aspects rated in the APACS tasks do not concern speech fluency, but rather mainly focus on core pragmatic elements. What seems to be compromised, and in relation with motor symptoms, is the ability to meet the interlocutor’s needs in conversation, possibly due also to reduced intentionality of engaging in a communicative exchange. As reported by some participants, the motor symptoms could trigger insecurity in being able to carry on a successful conversation, leading eventually to reduced discourse production (Snow and Douglas, 2017). Given the absence of a detailed assessment of language (due to time constraints), we cannot completely exclude a role of other linguistic abilities (e.g., phonological or grammatical) in influencing the production deficits in patients with PD. However, both qualitative and (albeit exploratory) quantitative analysis on the Interview items showed that (i) grammatical impairment was just one of the many facets of the deficits in discourse production shown by patients with PD, and indeed these patients did not show impairment in the Token Test and (ii) there was no evidence of phonological paraphasia. This might be indicative of discourse-pragmatic problems that are not “secondary,” i.e., caused by difficulties in structural aspects of language or by sensorimotor deficits (as reported, for instance in aphasia; see Perkins, 2003), but rather “genuine,” albeit possibly co-existent with difficulties in other language domains.

Concerning pragmatic skills in the comprehension modality, patients with PD showed worse performance than healthy controls in two tasks: Narratives and Humor. The Narratives task measures the ability to comprehend and to make inferences about different aspects of a narrative text, while the Humor task measures the ability to follow a story and to select the humoristic ending of that story among multiple choices (Arcara and Bambini, 2016). Interestingly, the impairment in comprehension was not generalized, as patients with PD did not show problems in the two other tasks targeting receptive aspects, that is Figurative Language 1 and 2, devoted to assess the ability to understand idioms, metaphors, and proverbs.

Why did patients with PD show problems in understanding narratives and humor? A potential explanation can be found considering the communalities between these tasks. Both Narratives and Humor include stories. It is possible, thus, that patients with PD show impairment whenever information about a story, including the monitoring of different aspects such as protagonists, relations, spatial and temporal elements, has to be kept “on-line” to make correct inferences. The hypothesis of a problem in the inferential aspects of attending a story is confirmed by the performance of patients with PD in the SET test (Story Based Empathy Test), a test of Theory of Mind with short stories described by pictures (Dodich et al., 2015). Patients with PD showed lower performance than healthy controls not only in typical ToM stories assessing the ability to understand intentions and emotions, but also in the control subscale of the SET test, which assesses the ability to understand causal inferences (e.g., a short scenario in which a person with a hat walks under a strong blowing wind, with several possible endings on what could happen to the hat). Given the difficulties also in the control scale, the low performance in SET should not be considered as necessarily related to Theory of Mind problems, but rather as linked to the difficulties in choosing the correct ending of a story. Interestingly, also previous studies on individuals who sustained a TBI reported problems in inferring from stories, not limited to mentalistic contents but extending to physical causality: these broader inferential difficulties were associated with pragmatic problems such as irony understanding (Martin and McDonald, 2005). Based on this evidence, we think that a general difficulty in inferring from stories may characterize pragmatic comprehension difficulties in PD.

Conversely, why did patients with PD show no impairment in figurative language tasks? There might be several reasons underlying this finding. First, this finding might capture a specificity of the communicative profile of patients with PD, which might be specifically impaired at the textual-inferential level (as in narratives and humor), rather than at the abstraction level (as in figurative language). Second, it might be that the impairment in figurative language is associated with global cognitive decline, rather than with PD. The finding from the study of Monetta and Pell (2007), where only patients with working memory impairment were impaired in metaphor comprehension, is consistent with this view. Here, we tested a sample of patients with variable cognitive skills (and the majority were not cognitively impaired), which might be the reason of the spared figurative language skills. We cannot rule out, however, that PD is associated with a difficulty with figurative language, but this is not captured by the APACS test and more fine grained tests (for instance with reading times) might detect a lower performance compared to controls.

Combining the findings on the various APACS tasks, the main problems in pragmatics associated with PD seem to lie at the discourse level, both in production (as the speech is not adequate with respect to the interlocutor’s needs, being underinformative and lacking verbal initiative) and in comprehension (as the understanding and the inferencing from narrative texts and humorous stories is impaired). It is interesting to note that the pattern of pragmatic disruption observed in PD is different from the patterns reported for other neurodegenerative diseases, such as ALS and MS, and for schizophrenia (Bambini et al., 2016a,b; Bosia et al., 2016; Carotenuto et al., 2018a), especially for what concerns comprehension. In ALS, MS, and schizophrenia, the impairment in APACS comprehension tasks appears to be more widespread, encompassing not only the Narratives and the Humor tasks but also figurative language tasks, which suggests a general impairment in pragmatic abilities, affecting both inferencing from text and inferencing abstract meanings from non literal-language. The pattern of comprehension difficulties that we observed in PD seems to be linked to the tasks where there is a discourse dimension (as in the Narratives task and in the stories of the Humor task), and, when compared to the patterns exhibited by other pathologies, seems to indicate a specific profile of pragmatic impairment in PD, which is definitely worth more discussion and more studies in the future.

The performance of patients with PD in pragmatic comprehension tasks showed several correlations with all the other variables taken into account in the analysis, with the highest number of significant correlations for Education, MoCA, the Token Test, SET subscales, and CRIq scales. The follow-up PCA analysis indicated that a single component was able to capture more than 50% of variance in all these scores. In other words, people who obtained a low score in one of these tasks obtained low scores in all the other tasks and, vice versa, people who obtained a high score in one of these tasks obtained high scores in all the other tasks. This suggests that most of these correlations with comprehension scores (see Table 3) could be considered (at least partly) as different facets of the same phenomenon. This pattern was similar for patients with PD and healthy controls: even if a qualitative inspection of the correlations in PD and in healthy controls showed some differences, when directly testing these differences no significant effects were found after correcting for multiple comparisons (but see Supplementary Material for full uncorrected *p*-values). This lack of robust differences in the correlations suggests that, in general, the pattern of association of cognitive and pragmatic abilities in patients with PD could be quantitatively different from healthy controls, rather than qualitatively.

The results of the Token Test deserve further consideration. Besides showing several significant correlations with almost all pragmatic tasks, the Token test showed the second highest Importance in Random Forest analysis with Pragmatic Comprehension as dependent variable. This result indicates that, even when considering several predictors and their interaction, the Token Test is strongly associated with Pragmatic Comprehension. Given that the Token Test is a test of basic aspects related to language comprehension, one could tentatively conclude that these more basic aspects are the cause of the pragmatic deficit. We believe that this explanation is not sound for several reasons: first, if this was the case, we would have found impaired performance in all language comprehension tasks (whereas in fact we did not find any impairment in Figurative Language 1 and Figurative Language 2); second, the PCA results suggest that Token Test, together with other tests, is part of a more global pattern of correlations (including also tests in which language comprehension is minimal if not absent, as in the SET task). A better explanation can be found considering that the Token Test does not only tap language comprehension but also general cognitive abilities and highly correlates with global tests such as MMSE (Agrell et al., 1995). So, the importance of the Token Test in pragmatic comprehension is better accounted for when considering that such test captures also general cognitive abilities. This interpretation is also supported by the fact that not only patients with PD, but also healthy controls showed an analogous pattern of correlations between the Token Test and scores on Pragmatic Comprehension, and that the Token Test showed quite strong associations (both in correlations and in Random Forests) also with Pragmatic Production.

Interestingly, we did not find any significant correlation between the pragmatic tasks and the Digit Span BW, although as a group patients with PD showed, as expected from literature, a lower performance in working memory tasks as compared to healthy controls (Gilbert et al., 2005). This result suggests that at least one major aspect of executive functioning (i.e., working memory) is not strictly related to the pragmatic profile of patients with PD. However, this should not be taken to imply that executive functions in general are not related to pragmatic abilities in PD, as several aspects of executive functioning were not included in our assessment and might be compromised in PD, for example set maintenance (Kehagia et al., 2010; D’Aniello et al., 2015; Holtgraves and Giordano, 2017), with potential associations with pragmatic skills.

The second main aim of the present study was to investigate the potential compensatory role of Cognitive Reserve on pragmatic abilities. It is important to stress that to be able to evaluate the compensatory role of Cognitive Reserve on pragmatics, as well as on any other cognitive ability, the ideal experimental design consists of a longitudinal study including brain data. Indeed, only studies including more than one measurement over time may actually investigate if a higher reserve is associated with a different time course of decline in the case of ongoing deterioration. In particular, the presence of data on brain structure or function (as from structural MRI or EEG) is necessary to assess if a different decline is associated with a different *Brain Reserve*, which is the ability of the brain to cope with damage (Barulli and Stern, 2013). In the present study, instead, we employed a cross-sectional approach (without longitudinal assessment), relying only on a questionnaire based on individual features that are known to be associated with the Cognitive Reserve (Stern, 2002; Cabeza et al., 2018). Although they are often treated as equivalent, Cognitive Reserve and Brain Reserve can be considered as two different (although mostly overlapped) constructs. For these reasons, we can hardly make any conclusion on the effect of Brain Reserve on pragmatic abilities, and any consideration on the effect of Cognitive Reserve on pragmatic abilities should be evaluated with cautions.

Despite these acknowledged limitations, and given the high cost of longitudinal studies including brain data, the present cross-sectional study may provide new insights, highlighting the potential dynamics of compensations across different stages of decline, and can be considered as preliminary evidence for shaping future studies. Indeed, several other studies used an analogous approach to identify variables that could be influenced by Cognitive Reserve (Sánchez et al., 2002; Corral et al., 2006; Roldán-Tapia et al., 2012; Mondini et al., 2016; Arcara et al., 2017; Ciccarelli et al., 2018).

In an initial analysis comparing Cognitive Reserve in patients with PD and healthy controls, we did not find differences in most of the scores of the CRIq scale (Nucci et al., 2012): the global score of CRIq and the two subscores (CRI-Education and CRI-WorkingActivity; Nucci et al., 2012). The two groups, however, showed a significant difference in the CRI-LeisureTime subscore. Rather than a problem in group matching, this difference might be related to the impact that PD has on the health-related quality of life of patients. By increasing their social embarrassment and isolation, the disease may reduce, in patients, those activities related to leisure time (Martinez-Martin, 1998; Karlsen et al., 2000). Coming to the relationship between Cognitive Reserve and pragmatics, since Cognitive Reserve is reported to be associated with better performances in high-order cognitive tasks, and since both are developed and enhanced through social life experiences (Tucker and Stern, 2011), we expected significant and high correlations between Cognitive Reserve measures and pragmatic tasks. In particular, we expected an association of education with figurative language comprehension (Champagne-Lavau et al., 2012). This expectation was confirmed for the pragmatic comprehension abilities, by both correlations and Random Forests. Both analyses evidenced a strong relationship between Cognitive Reserve and Pragmatic Comprehension scores. Specifically, all tasks and composite scores in the comprehension section of APACS showed significant correlations with Cognitive Reserve. Moreover, in the Random Forest predicting Pragmatic Comprehension, CRI-Total score was among the variables with highest Importance. Interestingly, the variable with highest Importance (i.e., the variable that best predicted the Pragmatic Comprehension score) was Education, which is not only a crucial demographic variable, but also part of Cognitive Reserve itself (Nucci et al., 2012; see also Arcara et al., 2017 for considerations on education). The finding of an effect of Education on Pragmatic Comprehension but not on Pragmatic Production is not surprising, considering that the two sections of the APACS test evaluate different aspects of the pragmatic competence. In particular, in APACS, comprehension tasks widely assess figurative language comprehension (which is known to be influenced by education; see for instance Champagne-Lavau et al., 2012), while production tasks mostly assess the ability to produce an appropriate discourse, which seems less tied to schooling.

This strong association between Cognitive Reserve variables and performance in Pragmatic Comprehension suggests that having a socially rich and active life-style may have a compensatory effect on the decline of pragmatic abilities associated with neurodegeneration, and may protect the ability to understand non literal speech as well as narrative texts. Importantly, as the correlation between Cognitive Reserve variables was similar also for healthy controls, it can be argued that similar protective mechanisms act in normal aging as well.

There are of course some limitations to the current study. The first one is the lack of structural or functional neuroimaging data. In the absence of this kind of data, we cannot draw strong inferences on the neural bases of the pragmatic impairment in PD. The well-known damage in fronto-striatal circuits in patients with PD is likely to represent the neural substrate of the observed impairment (Monetta and Pell, 2007; Monetta et al., 2009), but this disruption might as well be just the primary and more common cause of alteration in the widespread fronto-temporo-parietal networks associated with pragmatics (Bambini et al., 2011; Catani and Bambini, 2014). For instance, there is evidence that the pragmatic impairment in MS might be associated with functional connectivity of inferior parietal regions and the paracingulate cortex (Carotenuto et al., 2018b). Exploring the neuroanatomical basis of social communication problems would offer important information to gain a deeper understanding of the pragmatic profile of PD.

A second limitation concerns the sample of patients. It is important to underline that patients with PD may show several degrees of cognitive functioning (Biundo et al., 2014), spanning from being cognitively normal (PD-CNT), to Mild Cognitive Impairment (PD-MCI), to Dementia (PDD). Given the limited sample of the present study we could not investigate deeply the differences across these different groups. Only future studies can shed light on this issue (but see the Supplementary Material for an analysis restricted to PD-CNT participants).

Another limitation concerns the experimental design. To properly draw conclusions on the protective role of Cognitive Reserve, it would be necessary to run a longitudinal study and to observe a different trajectory in decline for high cognitive reserve patients compared to low cognitive reserve ones (Barulli and Stern, 2013). The cross-sectional design of the present study does not allow to test the effect of Cognitive Reserve directly, and compensatory affects are only inferred from correlations with Cognitive Reserve variables. The limitation of a design based on correlations is valid not only for CRIq but also for the other variables. Moreover, as an intrinsic problem of any correlational study, we also acknowledge that we may have missed other relevant variables in explaining the pattern of impairment in patients with PD. For example, we already mentioned that only one test related to executive functioning was included here. Neither we assessed the level of depression of patients, a condition that shows comorbidity with PD (Sagna et al., 2014). Depression, possibly related to the severity of symptoms, might negatively affect the production of discourse (Snow and Douglas, 2017). Despite all these limitations (common to all cross-sectional studies), this study highlighted several interesting associations between pragmatic scores and Cognitive Reserve scores, offering suggestions on which are the potentially relevant variables t hat can be examined in future longitudinal studies.

Finally, we claimed that PD is associated with problems in the discourse dimension, both in comprehension and in production, and that these problems seem “genuine,” i.e., not resulting from difficulties in other linguistic domains. Although we have evidence for this claim, we cannot fully rule out the possible role of difficulties in structural aspects of language, such as grammar. Future studies should combine a pragmatic assessment with a fine-grained language assessment, to derive stronger conclusions on the communicative profile of PD.

## Conclusion

In conclusion, our study showed that, relative to healthy controls, patients with PD showed a pragmatic language disorder that is mainly characterized by deficits in the dimension of discourse production and in inferring from narrative and humorous stories (but not in understanding figurative language).

Pragmatic aspects of production were mostly associated with the severity of motor symptoms, whereas performance in comprehension tasks was mostly associated with the global severity of cognitive impairment. The pattern of correlations was not significantly different between patients with PD and healthy controls, suggesting that the behavioral pragmatic pattern of patients with PD is quantitatively, rather than qualitatively, different from controls.

Importantly, we also showed for the first time that Cognitive Reserve (as measured with a questionnaire) can be strongly associated with pragmatics in PD, specifically with pragmatic comprehension abilities. This suggests that Cognitive Reserve may compensate the pragmatic impairment that may arise in patients with PD as the disease progresses. However, longitudinal studies are needed to corroborate this hypothesis.

Taken all together and compared with the results observed for other pathological conditions, these findings point to some degree of specificity in the pragmatic profile of PD. The present study, however, just scratched the surface of the investigation of social communication behavior in PD, an aspect poorly investigated up to now despite its fundamental importance for daily living. To our opinion, the most relevant questions for future research should tackle the following issues: what about other aspects of the pragmatic competence that were not considered in the APACS test, such as understanding irony or speech acts? Are they impaired or spared as metaphors, idioms, and proverbs? Is there a qualitatively different pattern of pragmatic impairment across different stages of cognitive decline in PD? Will a longitudinal study confirm the compensatory effect of Cognitive Reserve, and can an external intervention on activities associated with reserve improve the cognitive and pragmatic status of patients with PD?

## Ethics Statement

The study was approved by the Ethics Committee of the University of Padua (Italy).

## Author Contributions

SoM conceived the study, collected the data, and wrote the manuscript. SaM conceived the study and wrote the manuscript. MS and AM supervised the clinical data collection. VB interpreted the data, wrote the manuscript, and was responsible for theoretical and clinical pragmatic aspects. GA conceived the study, analyzed the data, and wrote the manuscript.

## Conflict of Interest Statement

The authors declare that the research was conducted in the absence of any commercial or financial relationships that could be construed as a potential conflict of interest.

## Footnotes

1 Specifically, the Working Activity section of CRIq provides a classification that takes into account the complexity of the job performed and its level of responsibility. This allows for more fine-grained distinctions as compared to the usual dichotomy white collar worker vs. blue collar worker.

2 We restricted this analysis to a limited number of scores because in PCA the number of variables that can be included is limited by the number of observations (i.e., in our case the number of patients).
