# Corrigendum: Pragmatic Language Disorder in Parkinson's Disease and the Potential Effect of Cognitive Reserve

**DOI:** 10.3389/fpsyg.2019.02201

**Published:** 2019-10-08

**Authors:** Sonia Montemurro, Sara Mondini, Matteo Signorini, Anna Marchetto, Valentina Bambini, Giorgio Arcara

**Affiliations:** ^1^Department of General Psychology, University of Padua, Padua, Italy; ^2^Human Inspired Technology Research Centre, University of Padua, Padua, Italy; ^3^Gruppo Veneto Diagnostica e Riabilitazione, Padua, Italy; ^4^Center for Neurocognition, Epistemology and Theoretical Syntax, University School of Advanced Studies IUSS, Pavia, Italy; ^5^IRCCS San Camillo Hospital, Venice, Italy

**Keywords:** Parkinson's disease, pragmatic abilities, Cognitive Reserve, communication, discourse, figurative language

In the original article, there were mistakes in Figures 3, 4, and 5 as published. The image reported as Figure 3 is the image that should be reported as Figure 4. The correct Figure 3 is missing from the paper. The image reported as Figure 4 is basically a duplicate of Figure 5 (note that the small differences between the two figures are normal, and due to the stochastic processes involved in the generation of the results used for the image). Figure 5 has a minor mistake in the position of x-axis labels.

As a consequence of these mistakes, the legends reported in the manuscript for Figures 3, 4, and 5 do not appropriately describe what is depicted. The correct Figures 3, 4, and 5 and their respective legends appear below.

**Figure 3:**
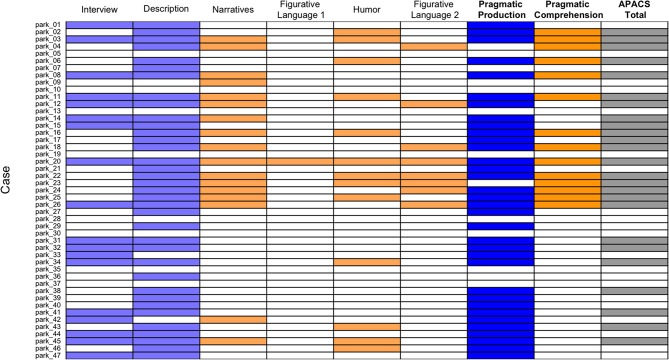
Performance below cut-off of Parkinson patients in pragmatic tasks and composite scores. The figure shows patients with PD who scored below cut-off (i.e., below 5th percentile of healthy control data) in the APACS tasks and in the three composite scores. Each row denotes a patient, whose case number is reported in the left part of the figure, consistently with **Supplementary Table S1**. Each column denotes a task or composite score. White cells indicate a performance equal to or above cut-off, whereas colored cells indicate a performance below cut-off. Light blue cells are used in the columns with the pragmatic tasks included in the Pragmatic Production score and dark blue cells are used for the Pragmatic Production score. Light orange cells are used in the columns of the pragmatic tasks included in the Pragmatic Comprehension score, and dark orange cells are used for the Pragmatic Comprehension score. Dark gray is used for APACS Total.

**Figure 4:**
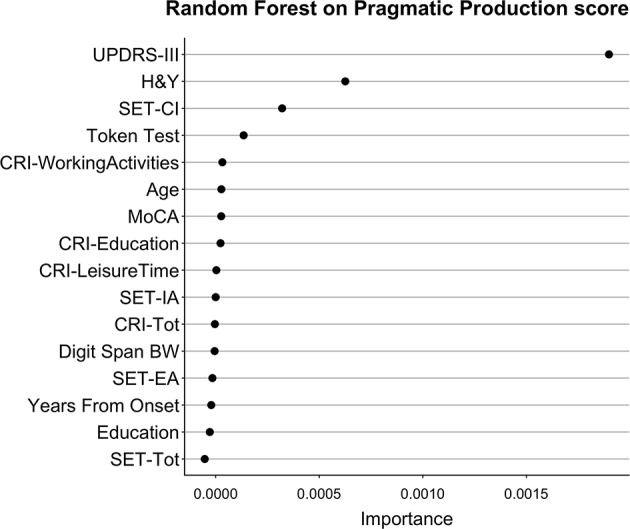
Importance of all variables in Random Forests with Pragmatic Production as dependent variable. The figure shows the Importance associated with each variable in the Random Forests with Pragmatic Production score from APACS as dependent variable. Variables are sorted from top to bottom according to Importance, so that the variables on top are the ones with the highest Importance.

**Figure 5:**
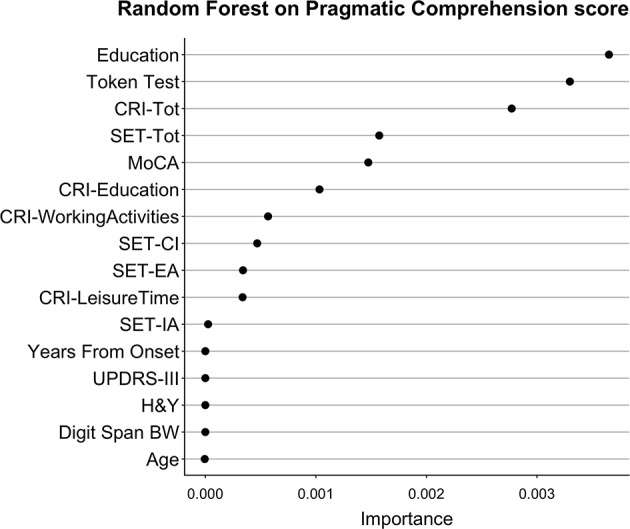
Importance of all variables in Random Forests with Pragmatic Comprehension as dependent variable. The figure shows the Importance associated with each variable in the Random Forests with Pragmatic Comprehension score from APACS as dependent variable. Variables are sorted from top to bottom according to Importance, so that the variables on top are the ones with the highest Importance.

The authors apologize for the errors and state that this does not change the scientific conclusions of the article in any way. The original article has been updated.

