# Supplementary material for: Pragmatic Language Disorder in Parkinson’s Disease and the Potential Effect of Cognitive Reserve
Source: Front Psychol. 2019 Jun 19;10:1220. doi: 10.3389/fpsyg.2019.01220 (PMC6593041; doi:10.3389/fpsyg.2019.01220)
Supplement: Supplementary file 1 [file Data_Sheet_1.pdf]

Supplementary Material

Pragmatic language disorder in Parkinson's Disease and the potential effect of Cognitive Reserve

Sonia Montemurro, Sara Mondini, Matteo Signorini, Anna Marchetto, Valentina Bambini, Giorgio Arcara*

***Corresponding author**:
Giorgio Arcara
Fondazione Ospedale San Camillo, IRCCS
Via Alberoni 70, 30126, Venezia, Italia
E-mail: giorgio.arcara@gmail.com

Table of Contents

Details on Patients with Parkinson's Disease 2
Details on Random Forest Analysis 3
Analysis on PD-CNT participants 5
Principal Component Analysis 6
Correlation figures 8
Comparison of correlations (patients with PD vs. healthy controls) 15
Details on comparison between patients with PD and Healthy Controls in Interview Task ... 21

Details on Patients with Parkinson's Disease

ID	Age	Education	Sex	Handedness	H&Y	UPRS-III	Years from Onset	MoCA
**Park_01**	48	10	F	R	3	46	9	28
**Park_02**	62	5	M	R	1	18	7	27
**Park_03**	81	3	M	R	3	35	7	20
**Park_04**	80	5	F	R	1	7	10	30
**Park_05**	76	13	M	R	1	8	7	30
**Park_06**	75	10	M	A	2	36	2	23
**Park_07**	71	5	F	R	2	22	9	26
**Park_08**	74	6	M	R	3	35	15	25
**Park_09**	72	5	F	R	3	24	10	30
**Park_10**	60	15	M	R	1	17	9	30
**Park_11**	69	8	M	R	2	33	10	21
**Park_12**	73	9	M	NA	2	33	3	16
**Park_13**	64	15	F	R	2	19	9	30
**Park_14**	74	18	M	R	3	37	16	30
**Park_15**	78	13	F	R	4	46	10	28
**Park_16**	72	18	F	R	2	40	4	25
**Park_17**	78	8	M	R	1	27	18	26
**Park_18**	78	5	F	R	3	33	9	24
**Park_19**	68	8	M	R	1	24	8	28
**Park_20**	64	5	M	R	3	44	10	16
**Park_21**	74	17	M	R	3	51	8	30
**Park_22**	78	5	M	R	1	23	3	22
**Park_23**	74	5	M	R	1	15	6	26
**Park_24**	73	8	M	R	2	28	8	16
**Park_25**	78	5	M	R	2	27	11	26
**Park_26**	70	5	M	R	3	47	10	8
**Park_27**	70	19	F	R	NA	NA	NA	18
**Park_28**	59	11	M	R	1	18	2	30
**Park_29**	88	18	M	R	3	48	4	28
**Park_30**	71	12	M	R	2	24	4	26
**Park_31**	82	10	M	R	3	47	3	26
**Park_32**	78	5	M	R	3	56	9	29
**Park_33**	78	18	M	R	2	40	5	29
**Park_34**	70	8	M	R	2	47	7	28
**Park_35**	78	18	M	R	3	52	14	28
**Park_36**	66	15	M	R	2	39	9	26
**Park_37**	59	12	F	R	1	30	9	29
**Park_38**	80	11	M	R	1	35	2	27
**Park_39**	74	17	F	R	3	46	8	26
**Park_40**	71	18	M	R	3	55	5	26
**Park_41**	59	8	F	R	4	60	3	25
**Park_42**	70	12	M	R	1	22	9	26
**Park_43**	78	11	M	R	2	39	11	25
**Park_44**	75	5	M	R	2	44	1	25
**Park_45**	69	9	M	R	4	57	5	26
**Park_46**	71	8	M	R	1	26	4	26
**Park_47**	74	13	M	R	1	28	3	24

S.1 Demographic and Clinical variables of patients with PD. The table reports the demographic and clinical variables of patients with PD. The first column reports the ID (also used in Figure 2), the second the Age (number of years), the third the Education (number of years), the fourth the Sex (Female or Male), the fifth the Handedness (Right, Left or Ambidextrous), the sixth the Hoehn and Yahr score (H&Y), the seventh the Unified Parkinson Disease Rating Scale score (UPDRS-III), the eighth the Years from Onset (YFO), the ninth the raw MoCA score. Missing values are reported as NA.

Details on Random Forest Analysis

The present paragraph reports a detailed explanation of the Random Forest Analysis included in the manuscript.

We decided to use Random Forests is to overcome the main limitations of the main pairwise correlations. In particular in pairwise correlations each pair of variables is considered separately and thus they are not able to capture interactions complex relationship across many variables, or redundancy.

Random Forests is a machine learning technique that seeks to find which predictors shows the highest association with a dependent variable. We decided to employ Random Forests (in place of the much more common multiple regressions) because of the potential limitations of using regressions in the current study. Multiple regressions have the main advantage of being very easy to interpret (James et al. 2013), but have the main disadvantage to require several assumptions[1]. Importantly for the present study, the number of predictors that one can meaningfully include in a multiple regression analysis strongly depends on the number of observations (in our case, the number of patients). A rule-of-thumb is to include one predictor every 15 observations (Harrel 2015). If the number of predictors is higher, the estimation of the effects may be unstable and untrustworthy. As our patient sample includes 47 individuals, this would limit our investigation to only three predictors (i.e., 47/15 = 3.13).

Random Forests (Breiman, 2001; Strobl, Boulesteix, Zeileis, & Hothorn, 2007; Strobl, Malley, & Tutz, 2009) represent an approach that does not suffer from the above-mentioned limitations: they can handle a high number of predictors with a low number observations; they also take into account interactions about variables, when the nature of relationship is not known; more generally, they can be used when the assumptions of linear regressions are not met. Random forests are a kind of statistical classifier deriving from simpler Classification And Regression Trees (CART). In CART a series of predictions, in the form of logical tests, are determined recursively by partitioning the data in smaller and smaller sets. The aim of CART partitioning is to minimize the mean square error (if the predicted variable is continuous) or the classification error (if the predicted variable is categorical) of the predictions. Basically, the results of a CART can be described by trees with a series of branches, each associated with a decision rule: if the condition is met, one proceeds to one side of the branch; if the condition is not met, one proceeds to the other side of the branch. This procedure is repeated until a single value is predicted (Baayen 2008). An interesting feature of CART is that they manage to capture, in a simple and intuitive way, interactions between predictors. A limitation of CART is that the initial split, which determines the whole tree, could be suboptimal. Furthermore, CART can suffer from data overfitting. Random Forests extends the potentialities of CART by generating several possible initial trees (a forest), including a random subset of predictors and observations (so not all predictors, and not all observations, i.e., the

[1] Among the most relevant assumptions of linear regressions are: 1) normal distribution of residuals; 2) homoschedasticity of residuals; 3) linear relationship between predictors and dependent variable; 4) non-collinearity between predictors. If these assumptions are not met, the results of a multiple regressions can be misleading and even wrong.

participants, are included to build each tree in the forest). Once several trees (typically hundreds) are built, it is possible to evaluate the overall performance of the forest and to assess the so-called "Importance" of each predictor. The Importance of a predictor indicates how much the overall predictions of the forests are hampered if the predictor is removed from the forest.

Even if Random Forests overcome some important limitations of CART and regressions, they have an important drawback: beside the Importance associated with each variable, they make "black-box" predictions and it is not easy to understand what is the real relationship between each predictor and the dependent variable. In other words, with random forests we can assess if a variable is a relevant predictor for a dependent variable, but we cannot easily assess how much this predictor is associated with the dependent variable. This is because several (even very complicated) interactions can explain the association.

Since, to our knowledge, this is the first study to investigate whether Cognitive Reserve, clinical and cognitive variables may predict pragmatic abilities in patients with PD, we decided to use the Random Forest approach to identify the most relevant variables. Importantly, only future studies could better characterize the results, focusing on fewer predictors and stronger hypothesis coming from previous evidence. Further and more specific analyses were not performed in this study to avoid circularity and the risk of overfitting the data.

We performed two Random Forest analyses, one with the Pragmatic Production score as dependent variable and one with the Pragmatic Comprehension score as dependent variable. In both analyses the predictors were: Demographic and Clinical variables (i.e., Age, Education, UPDRS-III, H&Y, Years from Onset), General Cognitive variables (i.e., MoCA, Token Test, Digit Span Backward, SET), and Cognitive Reserve. Random Forests were calculated using unbiased partitioning as implemented in the *cforest* function included in the *party* package (Strobl, Malley, & Gerhard, 2009; Strobl et al., 2009). Each forest consisted of 500 trees.

All statistical analyses were performed with R, release 3.3.2 (R core team, 2016).

Analysis on PD-CNT participants

The following section reports the results of t-tests on APACS restricted to patients with PD satisfying the criteria for PD-CNT group based on MoCA (i.e., MoCA > 25) (Biundo et al. 2014). Results are reported in Table S.2 and depicted in Figure S.1.

APACS tasks and composite scores	Mean Parkinson (SD)	Mean Controls (SD)	df	t-value	Cohen's d	p-value
**Interview**	36.06 (6.11)	38.82 (2.45)	74	-2.7	-0.64	0.014
**Description**	38 (6.23)	46.4 (1.99)	74	-8.5	-2	1.5e-11
**Narratives**	45.26 (7.09)	49.6 (4.33)	74	-3.3	-0.77	0.0032
**Figurative Language 1**	13.35 (1.68)	13.24 (2.09)	74	0,24	0.057	0.81
**Humor**	5.65 (1.78)	6.09 (1.24)	74	-1.3	-0.3	0.3
**Figurative Language 2**	22.26 (4.21)	21.09 (4.04)	74	1.2	0.28	0.3
**Pragmatic Production**	0.8 (0.11)	0.92 (0.03)	74	-7.1	-1.7	2.5e-09
**Pragmatic Comprehension**	0.81 (0.12)	0.83 (0.1)	74	-0.82	-0.19	0.47
**APACS Total**	0.81 (0.09)	0.88 (0.06)	74	-4.1	-0.97	0.00028

Table S.2 Descriptive statistics and results for t-tests comparing patients with PD (PD-CNT) and healthy controls in APACS. The table shows the results of t-test comparing patients with PD and Controls on the APACS tasks and three composite scores. The first column reports the name of the APACS task or composite score. The second and the third columns report the mean (and SD) of patients with PD and healthy controls. The fourth column reports the degrees of freedom associated with the t-test; the fifth column reports the t-values; the sixth column reports the Cohen's d. The seventh column reports the p-values (corrected with FDR method). The analysis shows the results restricted to the patients classified as PD-CNT.

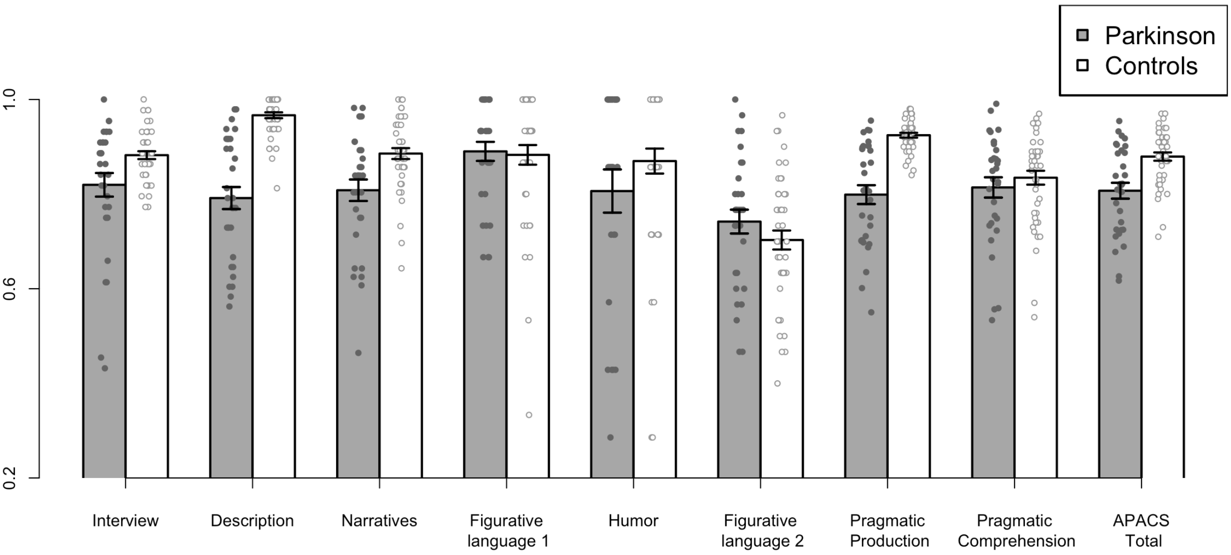

Figure S.1. Performance of patients with PD-CNT and Controls in APACS tasks and composite scores. The figure shows the performance of PD patients and healthy controls in the APACS tasks and composite scores, i.e., Pragmatic Production, Pragmatic Comprehension, and APACS Total. All raw scores were transformed in proportion (relative to the maximum obtainable score) before plotting. Gray bars indicate the mean performance of PD patients, whereas white bars indicate the mean performance of healthy controls. The small circles denote the scores for each participant (a small jitter was added in the x-axis to improve visibility of individual scores). The analysis shows the results restricted to the patients classified as PD-CNT.

Principal Component Analysis

To better understand the pattern of correlations of scores on APACS comprehension section with other scores we run two Principal Component Analyses (PCA), one for patients with PD, and one for healthy controls. The aim of this analysis was to find if the correlations were associated to a single underlying component and could be summarized by a single score. As in PCA the number of variables that can be included is limited by the number of observations (i.e., the number of patients), we restricted this analysis to the following variables: MoCA, Token Test, SET-Tot, CRI-Total, Pragmatic Comprehension. After removing participants with missing data, the PCA on patients with PD was run on a 44 x 5 matrix, while PCA on control was run on a 45 x 5 matrix. In both PCAs the first component explained a considerable amount of variance (62% for patients with PD, 58% for healthy controls). The inspection of loadings suggests that scores in the tests group together: participants who have high scores in a test, have high scores in all the other tests; vice versa, participants who have low scores in a test, tend to have low scores in all the other tests. This pattern was similar both for patients with PD and for healthy controls.

Results on loadings for Patients with PD and healthy controls are reported in Table S.3 and S.4.

Loadings of PCA on patients with PD					
	**PC1**	**PC2**	**PC3**	**PC4**	**PC5**
**MoCA**	-0.41	0.6	0.02	-0.68	0
**Token Test**	-0.46	0.39	-0.21	0.62	0.45
**SET-Tot**	-0.4	-0.57	-0.64	-0.28	0.17
**CRI-Total**	-0.43	-0.39	0.74	-0.07	0.34
**Pragmatic comprehension**	-0.52	-0.07	0.05	0.26	-0.81

Table S.3 Loadings of PCA on patients with PD. The table lists the loadings of the scores included in the PCA. Each column reports the loading of the score in the row, for each of the 5 components. The components explained the following proportion of variance PC1=0.62, PC2=0.16, PC3=0.11, PC4=0.08, PC5=0.04.

Loadings of PCA on healthy controls					
	**PC1**	**PC2**	**PC3**	**PC4**	**PC5**
**MoCA**	-0.5	0.01	-0.26	-0.37	0.74
**Token Test**	-0.42	0.41	0.76	0.26	0.11
**SET-Tot**	-0.44	0.36	-0.58	0.53	-0.24
**CRI-Total**	-0.37	-0.84	0.11	0.38	-0.01
**Pragmatic comprehension**	-0.49	-0.05	0.05	-0.61	-0.62

Table S.4 Loadings of PCA on healthy controls. The table lists the loadings of the scores included in the PCA. Each column reports the loading of the score in the row, for each of the 5 components. The components explained the following proportion of variance PC1=0.58, PC2=0.15, PC3=0.11, PC4=0.1, PC5=0.07.

Correlation figures

The figures in the following pages depict the scatterplots related to all correlations (see Table 4 and 5 of the main manuscript). Figures S.2, S.3, and S.4 report the scatterplots for patients with PD. Figures S.5, S.6, and S.7 report the scatterplots for healthy controls.

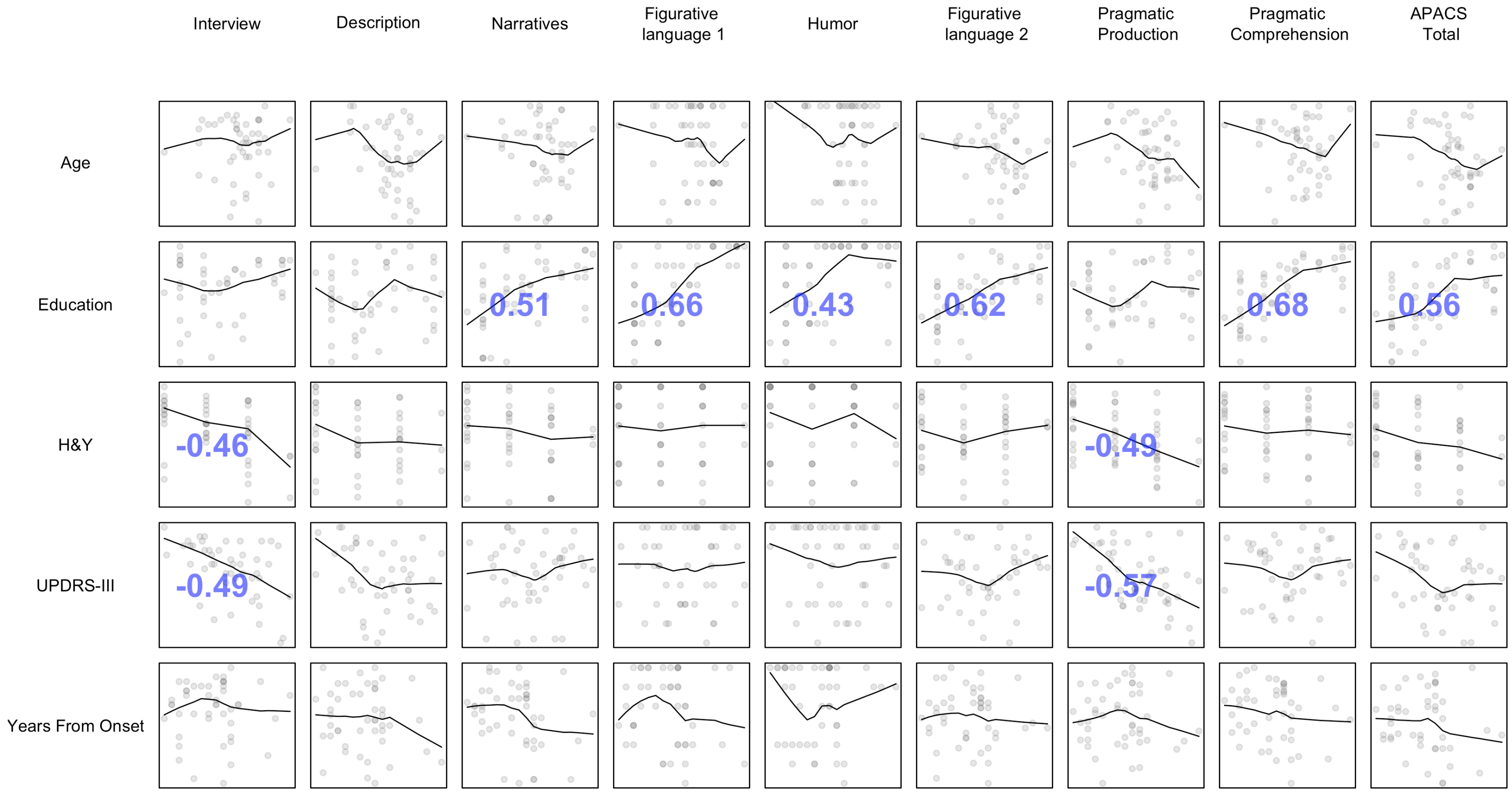

Figure S.2 Patients with Parkinson - Details of correlation between Demographic and Clinical Variables and APACS scores in patients with Parkinson. The figure shows all the scatterplots related to the correlations (see the manuscript for the full correlation matrix). Each dot represents a single subject. The numbers represent the Pearson's r correlation values. Only significant correlation values are reported. The depicted line is calculated through the *lowess* R function, which uses a locally-weighted polynomial regression to describe the trend of the data.

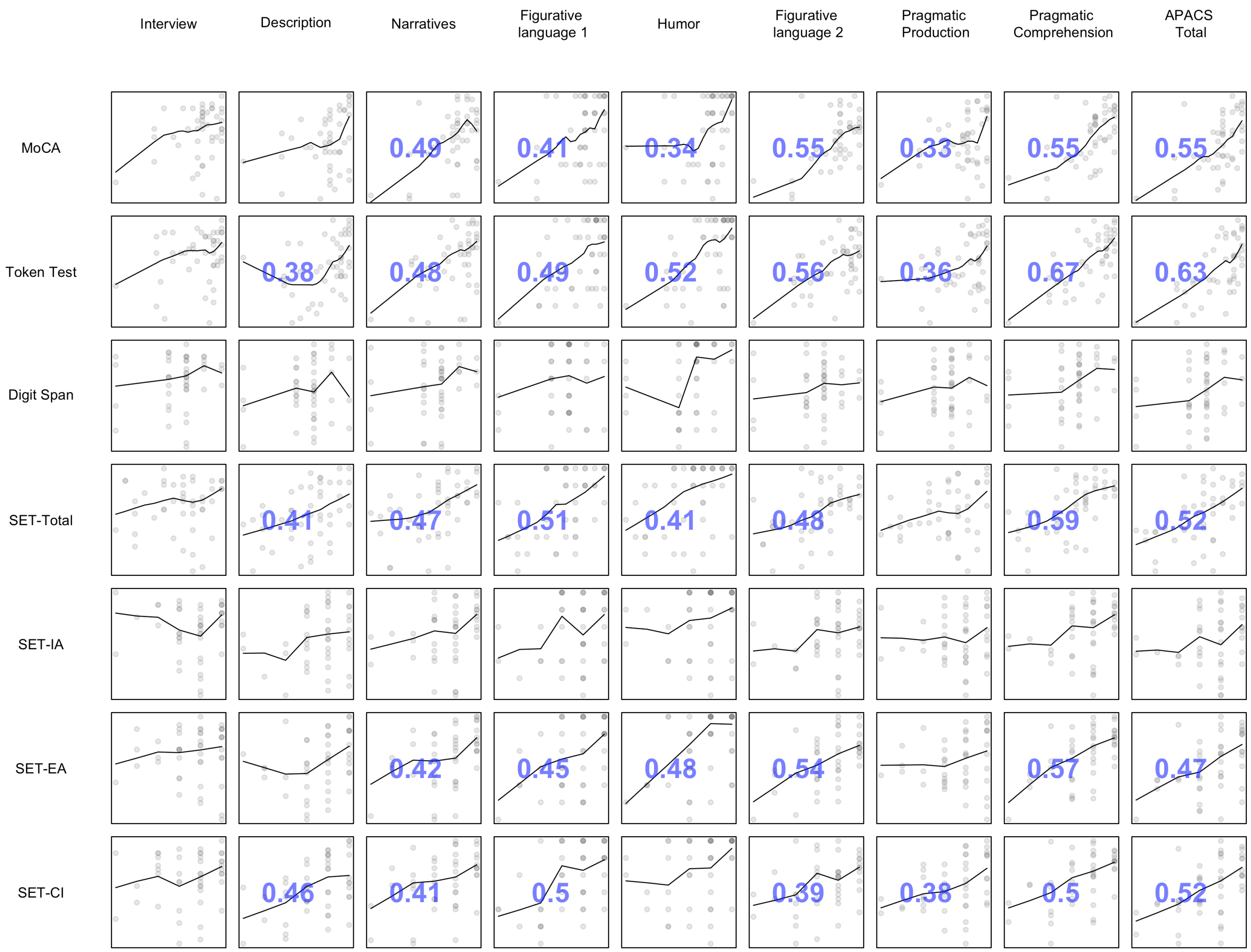

Figure S.3 Patients with Parkinson - Details of correlation between Cognitive Assessment Variables and APACS scores in patients with Parkinson. The figure shows all the scatterplots related to the correlations (see the manuscript for the full correlation matrix). Each dot represents a single subject. The numbers represent the Pearson's r correlation values. Only significant correlation values are reported. The depicted line is calculated through the *lowess* R function, which uses a locally-weighted polynomial regression to describe the trend of the data.

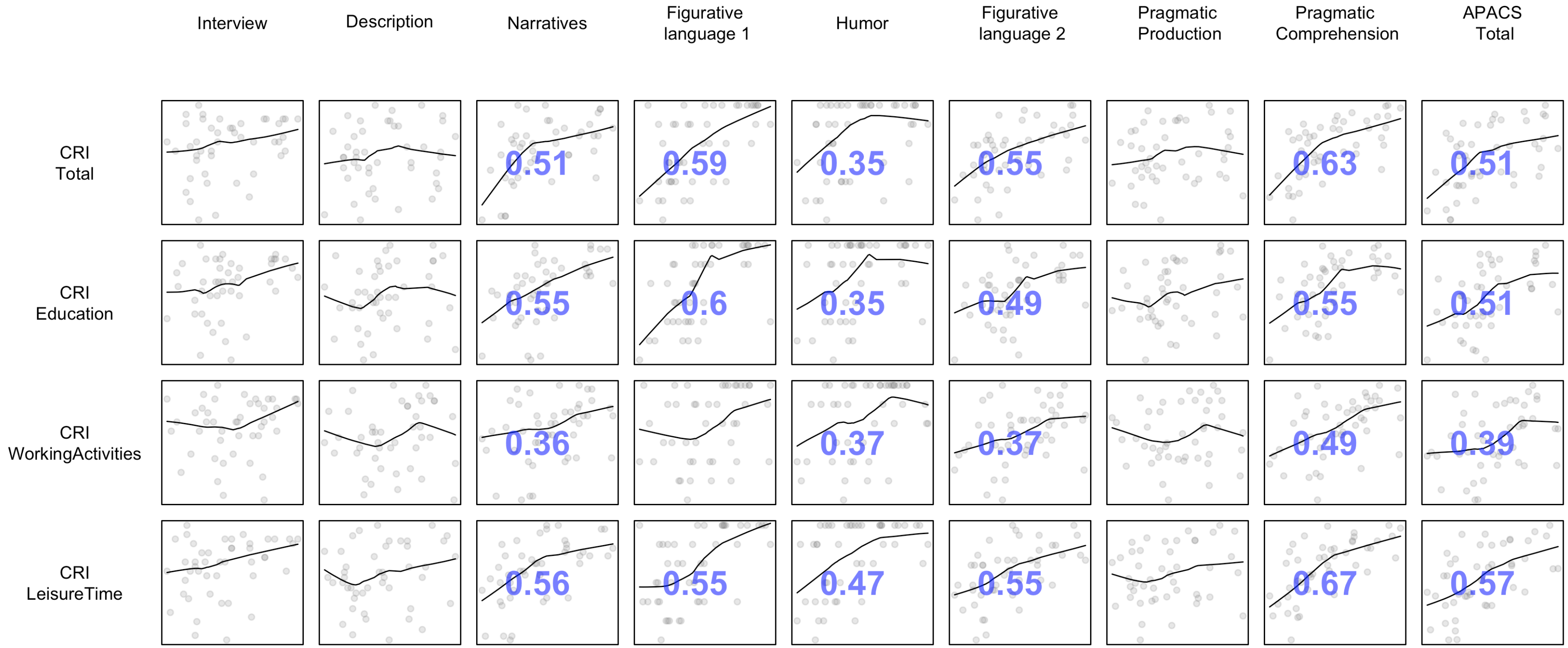

Figure S.4 Patients with Parkinson - Details of correlation between Cognitive Reserve Variables and APACS scores in patients with Parkinson. The figure shows all the scatterplots related to the correlations (see the manuscript for the full correlation matrix). Each dot represents a single subject. The numbers represent the Pearson's r correlation values. Only significant correlation values are reported. The depicted line is calculated through the *lowess* R function, which uses a locally-weighted polynomial regression to describe the trend of the data.

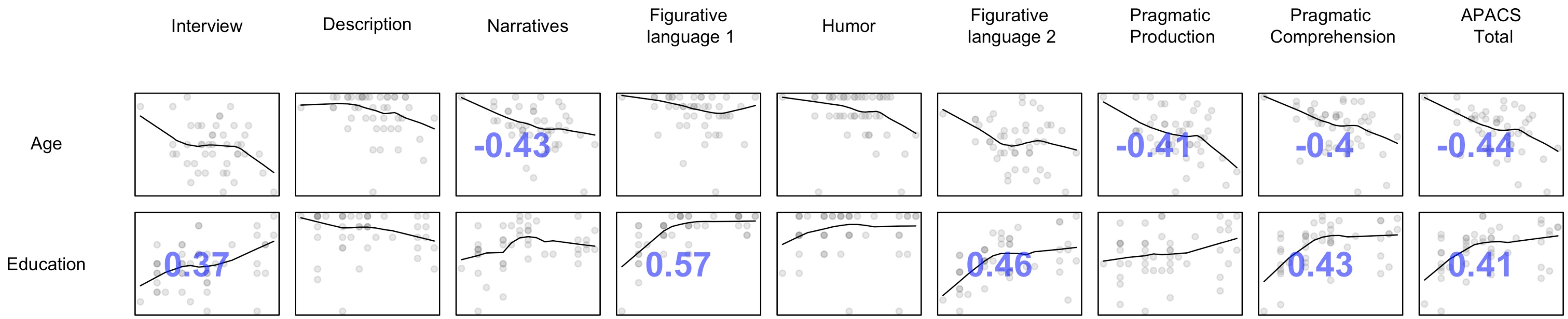

Figure S.5 Healthy controls - Details of correlation between Demographic and Clinical Variables and APACS scores in healthy controls. The figure shows all the scatterplots related to the correlations (see the manuscript for the full correlation matrix). Each dot represents a single subject. The numbers represent the Pearson's r correlation values. Only significant correlation values are reported. The depicted line is calculated through the *lowess* R function, which uses a locally-weighted polynomial regression to describe the trend of the data.

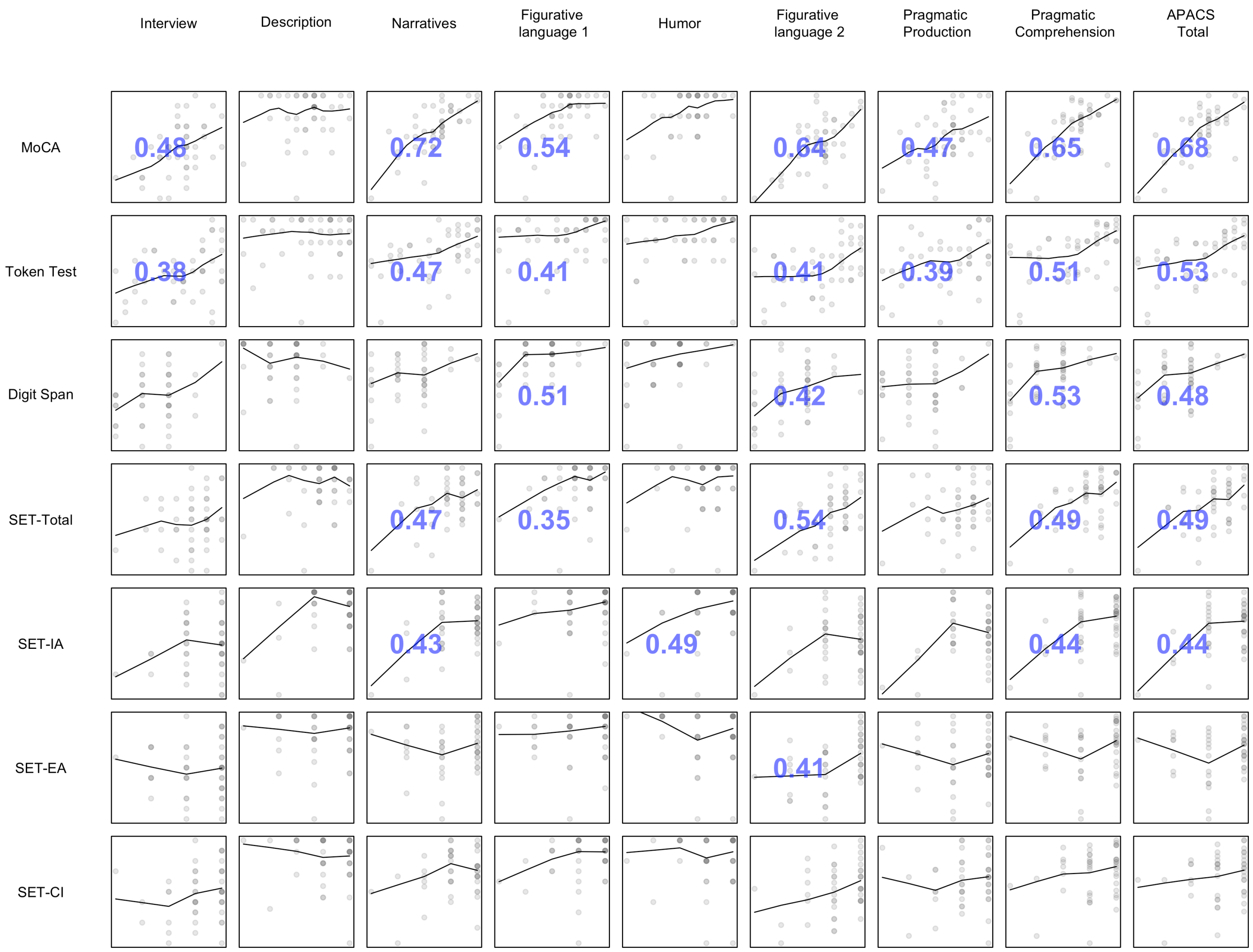

Figure S.6 Healthy controls - Details of correlation between Cognitive Assessment Variables and APACS scores in healthy controls. The figure shows all the scatterplots related to the correlations (see the manuscript for the full correlation matrix). Each dot represents a single subject. The numbers represent the Pearson's r correlation values. Only significant correlation values are reported. The depicted line is calculated through the *lowess* R function, which uses a locally-weighted polynomial regression to describe the trend of the data.

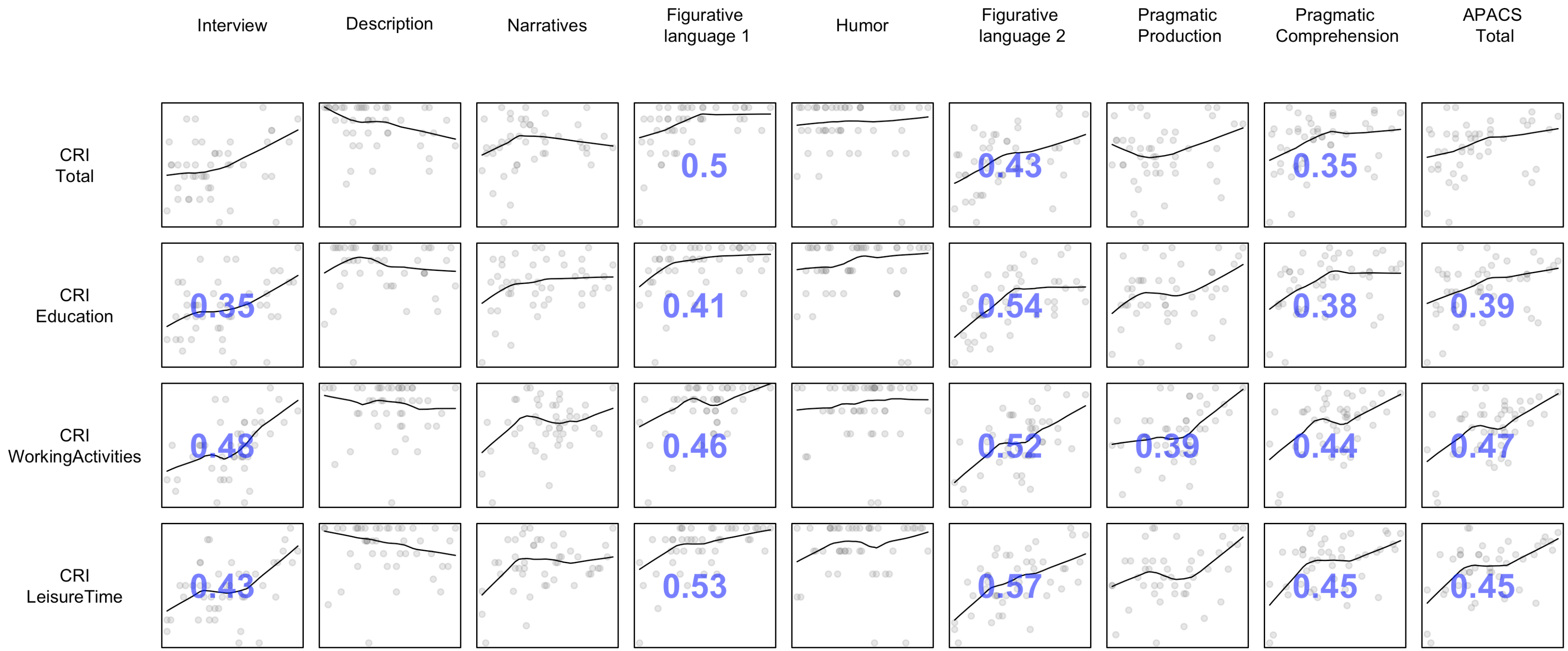

Figure S.7 Healthy controls - Details of correlation between Cognitive Reserve Variables and APACS scores in healthy controls. The figure shows all the scatterplots related to the correlations (see the manuscript for the full correlation matrix). Each dot represents a single subject. The numbers represent the Pearson's r correlation values. Only significant correlation values are reported. The depicted line is calculated through the *lowess* R function, which uses a locally-weighted polynomial regression to describe the trend of the data.

Comparison of correlations (patients with PD vs. healthy controls)

The following tables report all the comparisons between the correlation values of patients with PD and healthy controls. None of the results was significant after FDR correction (Benjamini and Hochberg 1995). Comparisons are sorted for decreasing order of uncorrected p-values.

APACS score	Other score	Parkinson	Healthy controls	N Parkinson	N controls	z	uncorrected p-value	p-value (FDR corrected)
**Humor**	**SET-EA**	0.48	-0.12	45	45	2.95	0.003	0.36
**Description**	**SET-CI**	0.46	-0.10	45	45	2.74	0.01	0.36
**Pragm comprehension**	**SET-EA**	0.57	0.15	45	45	2.24	0.03	0.59
**Humor**	**CRI-Tot**	0.47	0.03	45	45	2.18	0.03	0.59
**Figurative Lang 1**	**Digit Span BW**	0.11	0.51	46	45	2.04	0.04	0.59
**Humor**	**Education**	0.43	0.03	47	45	1.97	0.05	0.59
**Narratives**	**CRI-Education**	0.51	0.18	45	45	1.79	0.07	0.59
**Humor**	**CRI-Education**	0.35	-0.02	45	45	1.75	0.08	0.59
**Pragm comprehension**	**CRI-Education**	0.63	0.35	45	45	1.75	0.08	0.59
**Narratives**	**MoCA**	0.49	0.72	47	45	1.74	0.08	0.59
**Narratives**	**Age**	-0.09	-0.43	47	45	1.72	0.08	0.59
**Humor**	**CRI-WorkingAct**	0.35	0.00	45	45	1.70	0.09	0.59
**Pragm**	**Education**	0.68	0.43	47	45	1.69	0.09	0.59

APACS score	Other score	Parkinson	Healthy controls	N Parkinson	N controls	z	uncorrected p-value	p-value (FDR corrected)
comprehension								
Figurative Lang 1	SET-EA	0.45	0.11	45	45	1.68	0.09	0.59
APACS Tot	SET-EA	0.47	0.15	45	45	1.68	0.09	0.59
Humor	SET-IA	0.17	0.49	45	45	1.65	0.10	0.59
Interview	CRI-LeisureTime	0.16	0.48	45	45	1.64	0.10	0.59
Pragm comprehension	Age	-0.09	-0.40	47	45	1.53	0.13	0.59
Humor	SET-CI	0.31	-0.01	45	45	1.51	0.13	0.59
Narratives	CRI-Tot	0.56	0.30	45	45	1.51	0.13	0.59
Description	Token	0.38	0.07	46	45	1.51	0.13	0.59
Pragm comprehension	CRI-Tot	0.67	0.45	45	45	1.48	0.14	0.59
Interview	Age	0.00	-0.30	47	45	1.47	0.14	0.59
APACS Tot	SET-CI	0.52	0.25	45	45	1.45	0.15	0.59
Pragm production	CRI-LeisureTime	0.09	0.39	45	45	1.45	0.15	0.59
Description	CRI-Education	0.14	-0.17	45	45	1.44	0.15	0.59
Narratives	SET-EA	0.42	0.13	45	45	1.43	0.15	0.59
Humor	CRI-LeisureTime	0.37	0.08	45	45	1.43	0.15	0.59
Figurative Lang 2	Digit Span BW	0.14	0.42	46	45	1.42	0.16	0.59
Narratives	CRI-WorkingAct	0.55	0.30	45	45	1.41	0.16	0.59
Pragm comprehension	Digit Span BW	0.27	0.53	46	45	1.41	0.16	0.59
Pragm comprehension	SET-CI	0.50	0.25	45	45	1.40	0.16	0.59
Pragm production	SET-CI	0.38	0.11	45	45	1.32	0.19	0.65
Figurative Lang 1	SET-CI	0.50	0.26	45	45	1.27	0.20	0.65
Humor	Token	0.52	0.29	46	45	1.27	0.21	0.65
Narratives	Education	0.51	0.29	47	45	1.25	0.21	0.65

APACS score	Other score	Parkinson	Healthy controls	N Parkinson	N controls	z	uncorrected p-value	p-value (FDR corrected)
**Description**	**Education**	0.15	-0.11	47	45	1.24	0.21	0.65
**Description**	**CRI-Tot**	0.18	-0.08	45	45	1.22	0.22	0.65
**APACS Tot**	**Age**	-0.21	-0.44	47	45	1.22	0.22	0.65
**Description**	**SET-Tot**	0.41	0.17	45	45	1.22	0.22	0.65
**Description**	**Digit Span BW**	0.17	-0.09	46	45	1.21	0.23	0.65
**Interview**	**MoCA**	0.26	0.48	47	45	1.17	0.24	0.66
**APACS Tot**	**SET-IA**	0.22	0.44	45	45	1.17	0.24	0.66
**Pragm comprehension**	**Token**	0.67	0.51	46	45	1.15	0.25	0.66
**Figurative Lang 1**	**CRI-WorkingAct**	0.60	0.41	45	45	1.15	0.25	0.66
**Figurative Lang 2**	**Education**	0.62	0.46	47	45	1.09	0.27	0.69
**APACS Tot**	**Digit Span BW**	0.29	0.48	46	45	1.09	0.28	0.69
**APACS Tot**	**CRI-Education**	0.51	0.32	45	45	1.07	0.29	0.70
**Humor**	**SET-Tot**	0.41	0.22	45	45	1.00	0.32	0.73
**Description**	**SET-EA**	0.28	0.08	45	45	1.00	0.32	0.73
**Pragm comprehension**	**CRI-WorkingAct**	0.55	0.38	45	45	0.99	0.32	0.73
**APACS Tot**	**MoCA**	0.55	0.68	47	45	0.97	0.33	0.75
**APACS Tot**	**Education**	0.56	0.41	47	45	0.96	0.34	0.75
**Figurative Lang 2**	**Token**	0.56	0.41	46	45	0.92	0.36	0.76
**Figurative Lang 1**	**SET-Tot**	0.51	0.35	45	45	0.91	0.36	0.76
**Pragm production**	**SET-IA**	0.07	0.26	45	45	0.89	0.38	0.76
**Figurative Lang 2**	**CRI-LeisureTime**	0.37	0.52	45	45	0.87	0.39	0.76
**Figurative Lang 1**	**CRI-LeisureTime**	0.30	0.46	45	45	0.87	0.39	0.76
**Description**	**CRI-WorkingAct**	0.16	-0.02	45	45	0.83	0.40	0.76
**Humor**	**Age**	-0.12	-0.29	47	45	0.80	0.43	0.76
**Pragm production**	**MoCA**	0.33	0.47	47	45	0.78	0.44	0.76

APACS score	Other score	Parkinson	Healthy controls	N Parkinson	N controls	z	uncorrected p-value	p-value (FDR corrected)
**Figurative Lang 2**	**SET-EA**	0.54	0.41	45	45	0.78	0.44	0.76
**Interview**	**Education**	0.22	0.37	47	45	0.76	0.45	0.76
**Pragm production**	**Age**	-0.27	-0.41	47	45	0.76	0.45	0.76
**Interview**	**SET-IA**	-0.03	0.13	45	45	0.75	0.45	0.76
**Pragm comprehension**	**MoCA**	0.55	0.65	47	45	0.75	0.46	0.76
**Narratives**	**SET-IA**	0.29	0.43	45	45	0.73	0.46	0.76
**Pragm comprehension**	**SET-IA**	0.30	0.44	45	45	0.73	0.47	0.76
**Figurative Lang 2**	**CRI-Education**	0.55	0.43	45	45	0.73	0.47	0.76
**Figurative Lang 1**	**MoCA**	0.41	0.54	47	45	0.72	0.47	0.76
**Interview**	**CRI-Tot**	0.29	0.43	45	45	0.71	0.47	0.76
**APACS Tot**	**Token**	0.63	0.53	46	45	0.70	0.48	0.76
**Figurative Lang 1**	**Education**	0.66	0.57	47	45	0.70	0.48	0.76
**APACS Tot**	**CRI-Tot**	0.57	0.45	45	45	0.70	0.49	0.76
**Figurative Lang 2**	**Age**	-0.11	-0.25	47	45	0.69	0.49	0.76
**APACS Tot**	**CRI-WorkingAct**	0.51	0.39	45	45	0.69	0.49	0.76
**Description**	**CRI-LeisureTime**	0.13	-0.02	45	45	0.67	0.50	0.76
**Narratives**	**SET-CI**	0.41	0.28	45	45	0.66	0.51	0.76
**Pragm comprehension**	**SET-Tot**	0.59	0.49	45	45	0.65	0.52	0.76
**Figurative Lang 2**	**MoCA**	0.55	0.64	47	45	0.64	0.52	0.76
**Description**	**MoCA**	0.29	0.16	47	45	0.64	0.52	0.76
**Interview**	**Token**	0.26	0.38	46	45	0.61	0.54	0.77
**Figurative Lang 1**	**CRI-Education**	0.59	0.50	45	45	0.60	0.55	0.77
**Humor**	**MoCA**	0.34	0.22	47	45	0.59	0.55	0.77
**Figurative Lang 1**	**Age**	-0.14	-0.26	47	45	0.57	0.57	0.78
**Pragm production**	**CRI-Tot**	0.21	0.32	45	45	0.54	0.59	0.80
**Interview**	**Digit Span**	0.18	0.29	46	45	0.54	0.59	0.80

APACS score	Other score	Parkinson	Healthy controls	N Parkinson	N controls	z	uncorrected p-value	p-value (FDR corrected)
	BW							
Interview	SET-Tot	0.07	0.18	45	45	0.49	0.62	0.83
Pragm production	SET-EA	0.15	0.05	45	45	0.48	0.63	0.83
Figurative Lang 1	Token	0.49	0.41	46	45	0.46	0.64	0.83
APACS Tot	CRI-LeisureTime	0.39	0.47	45	45	0.46	0.65	0.83
Narratives	Digit Span BW	0.24	0.32	46	45	0.44	0.66	0.83
Description	SET-IA	0.23	0.30	45	45	0.36	0.72	0.90
Pragm production	Education	0.17	0.25	47	45	0.36	0.72	0.90
Figurative Lang 2	SET-Tot	0.48	0.54	45	45	0.34	0.73	0.90
Narratives	CRI-LeisureTime	0.36	0.29	45	45	0.31	0.75	0.90
Pragm comprehension	CRI-LeisureTime	0.49	0.44	45	45	0.31	0.75	0.90
Description	Age	-0.23	-0.30	47	45	0.31	0.76	0.90
Figurative Lang 2	CRI-WorkingAct	0.49	0.54	45	45	0.30	0.76	0.90
Figurative Lang 2	SET-CI	0.39	0.33	45	45	0.30	0.77	0.90
Interview	CRI-WorkingAct	0.30	0.35	45	45	0.23	0.82	0.95
Pragm production	CRI-WorkingAct	0.25	0.29	45	45	0.21	0.84	0.95
Interview	SET-CI	0.15	0.19	45	45	0.20	0.84	0.95
Interview	SET-EA	0.04	-0.01	45	45	0.19	0.85	0.95
Figurative Lang 2	CRI-Tot	0.55	0.57	45	45	0.17	0.86	0.95
Figurative Lang 1	CRI-Tot	0.55	0.53	45	45	0.17	0.87	0.95
APACS Tot	SET-Tot	0.52	0.49	45	45	0.17	0.87	0.95
Pragm production	Token	0.36	0.39	46	45	0.15	0.88	0.95
Interview	CRI-Education	0.26	0.29	45	45	0.14	0.89	0.95
Figurative Lang 1	SET-IA	0.24	0.21	45	45	0.13	0.90	0.96
Pragm production	CRI-Education	0.17	0.15	45	45	0.10	0.92	0.97

APACS score	Other score	Parkinson	Healthy controls	N Parkinson	N controls	z	uncorrected p-value	p-value (FDR corrected)
**Pragm production**	**Digit Span BW**	0.17	0.19	46	45	0.09	0.93	0.97
**Pragm production**	**SET-Tot**	0.26	0.24	45	45	0.07	0.94	0.98
**Figurative Lang 2**	**SET-IA**	0.21	0.20	45	45	0.06	0.95	0.98
**Narratives**	**Token**	0.48	0.47	46	45	0.05	0.96	0.98
**Humor**	**Digit Span BW**	0.31	0.31	46	45	0.03	0.98	0.99
**Narratives**	**SET-Tot**	0.47	0.47	45	45	0.01	0.99	0.99

Table S.5 Comparison of Correlation values between patients with PD and healthy controls. The table reports the comparison of correlation values for PD and healthy controls. The first two columns report the two variables involved in the correlation. The remaining columns report the details on the statistics comparing patients with PD and healthy controls in that correlation.

Details on comparison between patients with PD and Healthy Controls in Interview Task

APACS subtest	w	p	p FDR	Median Parkinson	Median Controls	sig
**Anomia**	970	0.38	0.532	2	2	
**Agrammatism**	1600	0.0000092	0.0000483	2	1	*
**Phonemic Paraphasias**	1000	0.87	0.961578947	2	2	
**Semantic Paraphasias**	900	0.087	0.140538462	2	2	
**Circomlocutions**	1000	0.73	0.901764706	2	2	
**Repetitions/Passpartout expressions**	1500	0.000069	0.0002898	2	1	*
**Incomplete sentences**	1000	0.79	0.921666667	2	2	
**Echolalia**	1200	0.06	0.105	2	2	
**Coprolalia**	1100	NA	NA	2	2	
**Difficulty with Yes/No answers**	880	0.012	0.022909091	2	2	*
**Underinformativeness**	910	0.1	0.15	2	2	
**Overinformativeness**	1100	1	1	2	2	
**Lack of verbal initiative**	760	0.00091	0.00273	2	2	*
**Lack or wrong use of cohesive ties**	1100	0.55	0.721875	1	1	
**Lack of discourse referents**	740	0.0011	0.0028	2	2	*
**Wrong order of narrative elements**	1100	0.96	1	2	2	
**Abrupt topic shifts**	760	0.00016	0.00056	2	2	*
**Altered speech rate**	610	0.0000011	0.0000077	2	2	*
**Altered intonation**	490	0.000000032	0.000000336	1	2	*
**Lack of eye contact**	810	0.0012	0.0028	2	2	*
**Fixed facial expression**	320	9.7E-12	2.037E-10	1	2	*
**Abuse of compensatory gesture**	850	0.0048	0.01008	2	2	*

Table S.6 Comparison of patients with PD and healthy controls in the Interview Task. The table reports the comparison between patients with PD and healthy controls in the Interview Task of APACS (Arcara and Bambini 2016). Specifically, the table contains: the Interview item (first column), the W of the Wilcoxon Test for unpaired samples (second column), the uncorrected p-value (third column), the FDR corrected p-value (fourth column), the median of patients with PD (fifth column), the median of healthy controls (sixth column). The last column shows an asterisk if the p-value (after FDR correction) was below 0.05.
